# Gene–radon interaction effects on lung cancer risk: validation of susceptibility loci using archived DNA from German Uranium miners

**DOI:** 10.1186/s41021-026-00365-z

**Published:** 2026-10-26

**Authors:** Albert Rosenberger, Irina Bonzheim, Falko Fend, Beate Hochstrat, Georg Johnen, Alexander Brik, Peter Rozynek, Heike Bickeböller, Simone Mörtl, Maria Gomolka

**Affiliations:** 1 Department of Genetic Epidemiology, University Medical Center, Georg-August- University Göttingen, Göttingen, Germany; 2 Institute of Pathology and Neuropathology, University Hospital Tuebingen and Comprehensive Cancer Center South West, Tübingen, Germany; 3 Section Radiation Epidemiology, Federal Office for Radiation Protection, Oberschleißheim, Germany; 4 Institute for Prevention and Occupational Medicine of the German Social Accident Insurance, Institute of the Ruhr University Bochum (IPA), Bochum, Germany; 5 Section Radiation Biology, Federal Office for Radiation Protection, Oberschleißheim, Germany

**Keywords:** Arsenic, Lung neoplasms, Uranium, Genotype, Radon, DNA, Miners, Occupational exposure

## Abstract

**Background:**

Radon exposure increases lung cancer risk, shown in studies on indoor exposure (low exposure) and uranium miners (high exposure). Genome-wide and candidate gene studies have identified DNA variants that interact with occupational radon to modify lung cancer risk.

**Method:**

Highly fragmented DNA samples of 611 individuals were genotyped using next-generation sequencing. DNA variant associations could be assessed in 526 out of 611 WISMUT uranium miners with lung cancer. The relative risk of lung cancer per allele (marginal effect) as well as its dependence on radon exposure (gene-environment (GxE) interaction) could be estimated from the enrichment of one allele compared to the distribution of genotypes expected under the HWE assumption (Hardy-Weinberg equilibrium). Exposure to gamma radiation, long-lived radionuclides, fine dust, silicate dust and arsenic was considered in the same way.

**Results:**

For markers from six (*UBE2U*,* CDKN2A*,* SIRT1*,* NAV2*,* ST8SIA2*,* TP53*) of the 10 genes examined, a significant modification of the genetic relative risk for lung cancer due to mining-specific radon exposure [in WLM] was observed.

**Conclusion:**

Radon exposure may be the main cause of some of the observed GxE interactions involving *UBE2U*,* CDKN2A* and *SIRT1*, but it may not be the only one. This conclusion is supported by the function of these genes, as based on relevant literature. For another marker of the gene *SOX5*, a GxE interaction can presumably be attributed to exposure to arsenic. For the remaining genes, no single environmental factor (radon, dust or arsenic) can unambiguously be identified as sole cause of the GxE interaction.

**Clinical trial number:**

Not applicable.

**Supplementary Information:**

The online version contains supplementary material available at https://doi.org/10.1186/s41021-026-00365-z.

## Introduction

Radon-222 (Rn-222) is a radioactive gas formed naturally from uranium-238 decay. Its concentration depends on uranium content in rocks [1]. With a half-life of 3.8 days, it decays into short-lived solid radioactive progeny. As a gas, it diffuses through soil and accumulates in enclosed spaces, such as mines and houses. Humans are mainly exposed through inhalation. During decay into short-lived radioactive progeny products such as polonium-218 (Po-218) and polonium-214 (Po-214), it emits high-energy alpha particles, along with beta and gamma radiation, contributing to cumulative radiation exposure. Radon exposure is the main source of ionizing radiation for human beings, particularly in radon-prone areas.

Alpha particles, the main exposure source, release high energy over short distances, strongly ionising and damaging DNA. Though poorly penetrating, their intense ionisation events cause severe direct damage to DNA and by ionizing water molecules also oxidative stress by generated free radicals. Inhaled radon and its progeny deposit on bronchial epithelium, where alpha particles primarily harm respiratory cells, initiating and promoting lung carcinogenesis through cytotoxic and genotoxic effects. These lead to far-reaching molecular DNA changes, such as double-strand breaks, mutations, deletions, and chromosomal rearrangements potentially initiating cancer development [2–5]. Despite this evidence, the exact mechanism for radon causing lung carcinogenesis is largely unknown.

Several studies have demonstrated an increased radon-induced risk of lung cancer (LC) in relation to indoor exposure (low dose) and occupational exposure in uranium miners (high dose) [1, 6–8].

Although smoking is by far the most significant risk factor for lung cancer, an individual genetic predisposition, spread across more than 45 susceptibility genomic loci, has been identified [9]. However, a genome-wide study was only able to identify a few single nucleotide polymorphisms (SNPs) that influence the risk of lung cancer in interaction with occupational radon exposure. The study included radon-exposed WISMUT miners, both with and without lung cancer (collected by the Federal Office for Radiation Protection, BfS), as well as lung cancer patients and control subjects from the general population with no occupational exposure to radon (collected by the International Lung Cancer Consortium, ILCCO) [10]. Radon gene interactions were observed at additional loci by other studies with candidate gene approaches and of much smaller sample size, e.g., for GSTM1 [11], IL-6 and *SIRT1* [12], or MGMT [13]. However, none of these radon-specific associations/interactions observed in individual studies have yet been replicated.

The aim of this project was to validate or replicate reported interactions of radon with genomic loci noted as gene-environment interaction GxE) with respect to lung cancer risk in miners. Reported GxE refers to findings of the genome-wide interaction search [10] as well as results of the other studies. Replication was carried out in an independent series of 611 DNA samples from uranium miners suffering from lung cancer stored in the German Uranium Miners’ Biobank, which were provided by the Federal Office for Radiation Protection (BfS). The biospecimens of these samples consisted of highly fragmented DNA find example figure of DNA in agarose gel in Additional file 1), isolated from archived formalin-fixed paraffin-embedded (FFPE) normal lung tissue [14, 15]. As nucleic acid integrity can decrease depending on freeze and thaw cycles during storage, and non-buffered formalin was used at the time of preservation, the isolated DNA quantity and quality limited strongly the number of markers amenable to genotyping.

## Methods

### Selection of markers for genotyping

Based on a comprehensive literature search in 2018, a list of 34 genes was compiled for which associated genomic loci have been reported to interact with radon in relation to lung cancer risk. These genes were evaluated and prioritised according to association, function and expression [16–19]. These genes were either associated with radon-induced LC, known as lung cancer risk-genes or considered relevant to lung cancer pathogenesis. Although this prioritisation was based on objective characteristics, it was, to a certain extent, subjective due to the weighting given to these characteristics. Details are laid out in the Additional file 1. The following 10 genes (in the given order, listed in Table 1) were prioritised for validation: *FTO*, *UBE2U*, *CUBN*, *ST8SIA2*, *SOX5*, *TP53*, *CD163L1*, *CDKN2A*, *NAV2* and *SIRT1*. Two to four markers per gene were selected for typing, covering the affected regions in total 29 markers). The selection was based on the marker-specific significance (p-value) of a radon x gene interaction [10], the linkage disequilibrium (LD) between the markers and the frequency of the rarer allele minor allele frequency MAF, > 5%).

**Table 1:** External information on the markers selected to be genotyped

No.	marker	chr.	position	MAF	major allele	minor allele	gene	selection reason
1	rs7545208	1	64 665 046	33.9%	A	C	*UBE2U*	LD-Sig.
2	rs7526950	1	64 684 038	22.9%	C	T	*UBE2U*	LD-Sig.
3	rs10889478	1	64 804 890	48.1%	T	C	*UBE2U*	LD-Sig.
4	rs1908635	1	64 805 503	17.2%	C	A	*UBE2U*	significance
5	rs7023680	9	21 816 573	47.0%	A	G	*CDKN2A*	significance
6	rs3731217	9	21 984 661	14.3%	A	C	*CDKN2A*	significance
7	rs10965227	9	22 081 796	23.3%	A	G	*CDKN2A*	significance
8	rs73596224	10	17 005 956	6.6%	C	A	*CUBN*	significance
9	rs60918373	10	17 046 641	15.2%	T	A	*CUBN*	LD-Sig.
10	rs7095324	10	17 250 900	36.3%	G	T	*CUBN*	significance
11	rs10823112	10	69 670 816	6.0%	A	G	*SIRT1*	LD-Sig.
12	rs10762183	10	69 713 297	32.0%	A	C	*SIRT1*	LD-Sig.
13	rs7129281	11	19 265 226	26.8%	C	T	*NAV2*	significance
14	rs11025138	11	19 466 820	30.2%	T	C	*NAV2*	LD-Sig.
15	rs4756999	11	19 468 769	33.5%	C	A	*NAV2*	significance
16	rs7137426	12	7 382 236	37.0%	G	T	*CD163L1*	significance
17	rs7970379	12	7 451 788	5.0%	A	G	*CD163L1*	LD-Sig.
18	rs7302538	12	7 499 479	5.5%	C	T	*CD163L1*/ ACSM4	significance
19	rs1353793	12	24 190 988	45.0%	G	T	*SOX5*	LD-Sig.
20	rs7978583	12	24 244 724	46.5%	T	C	*SOX5*	significance
21	rs4777965	15	92 898 565	38.5%	T	C	*ST8SIA2*	LD-Sig.
22	rs1455777	15	92 988 741	49.9%	G	A	*ST8SIA2*	LD-Sig.
23	rs3784722	15	93 006 088	36.8%	A	G	*ST8SIA2*	significance
24	rs8061518	16	53 861 024	33.9%	A	G	*FTO*	LD
25	rs6499657	16	53 988 978	15.8%	G	C	*FTO*	significance
26	rs1111483	16	54 000 907	48.1%	A	C	*FTO*	LD
27	rs17886268	17	7 592 802	3.9%	C	T	*TP53*	significance
28	rs7141	17	7 614 601	36.6%	A	G	*TP53*	significance
29	rs7221056	17	7 711 126	33.2%	C	T	*TP53*	significance

Chr. chromosome; position on chromosome in [bp] according to Genome Reference Consortium Human Build 37 (GRCh37); MAF “minor allele frequency” according to SNPNexus [20]; gene closest (assigned) gene according to SNPNexus. Selection reason: LD-Sig. observed interaction and LD with most significant marker, LD LD with most significant marker

### Genotyping

#### Determination of the genotype by NGS

Integrity of the biospecimens and the DNA quantity were checked before *g*enotypes were determined by next generation sequencing (NGS). To establish the NGS custom panel, 15 normal-tissue DNA samples with severe DNA fragmentation (100–200 base pairs), taken from the archive of the Institute of Pathology in Tübingen, were analysed.

A custom panel was designed for targeted analysis of 29 markers, optimized for highly degraded DNA with amplicon lengths up to 140 bp, with the Ampliseq Designer of Thermo Fisher Scientific (Waltham, MA, USA) (see Additional file 1). Amplicon library preparation, quantification (LightCycler 480), and sequencing were performed manually and automatically using Ion Chef and Ion GeneStudio S5 Prime systems, following manufacturer protocols (AmpliSeq Library Kit v2.0, Ion 510/520/530 & 540 Kits). DNA concentrations in the WISMUT samples were measured via Qubit (Thermo Fisher Scientific). Genotyping was conducted with as little as 10 ng of DNA. DNA samples were mixed with AmpliSeq HiFi Mix and the primer pool. PCR amplification was carried out in a thermocycler (Eppendorf, Hamburg, Germany) under the following conditions: initial denaturation at 99 °C for 2 min, followed by cycles of 99 °C for 15 s and 60 °C for 4 min. Following amplification, primer sequences were partially digested using FuPa reagent, and barcoded sequencing adapters (Ion Xpress Barcode Adapters, Thermo Fisher Scientific) were ligated to the amplicons. Libraries were subsequently purified with AMPure XP magnetic beads (Beckman Coulter, Brea, CA, USA) and quantified by qPCR on a LightCycler 480 Instrument (Roche, Basel, Switzerland) using the Ion Library TaqMan Quantitation Kit (Thermo Fisher Scientific). Quantified libraries were pooled and subjected to clonal amplification and enrichment on Ion Sphere particles using the Ion Chef system. Sequencing was then performed on an Ion GeneStudio S5 Prime instrument using Ion 530 or 540 chips. The feasibility of typing was tested in advance using another 35 pilot cases from the archive.

Initial genotyping of WISMUT samples showed poor DNA quality. To prevent amplification of fixation artefacts, PCR cycles were reduced from 25 to 26 to 22, and DNA amount was tripled if possible. Although more clear results were obtained, most samples remained insufficiently amplifiable. Thus, PCR cycles were increased back to 26, and genotyping repeated were possible. Therefore, NGS was performed with three different settings. A bioinformatics analysis (sequence generation, raw data processing, quality control, sample assignment, and file creation) was performed using Torrent Suite Software 5.16. Variant calling against the hg19 human genome was done with the variantCaller plugin.

A single genotype was coded by NGS with values between 0 and 100, where 0 stand for no copy of the reference allele (homozygous), 50 for 1 copy (heterozygous) and 100 for 2 copies (homozygous).

#### Translation of the genotype doses into genotype probabilities

Before testing the G×E interaction statistically, genotype doses were converted to genotype probabilities using the flexible parametric FLDSf function, transforming doses 0–50 and 100–50 to heterozygosity probabilities (0–1) under symmetric assumptions [21]. This function was integrated into the G-model describing genotype distributions [g₀ g₁ g₂] among “cases” (details given in the Additional file 1). Parameters were estimated by maximizing likelihood under HWE, allowing deviations due to genetic relative risk for lung cancer (RR, by marker, case-only design) and excess heterozygosity (accounting for hidden relatedness, population structure, or genotyping errors). This was applied per NGS setting and marker. Of 87 theoretical clusters (29 markers x 3 NGS-settings), only 12 showed strong similarity (nearest centroid sorting via PROC FASTCLUS, SAS^©^).

### Phenotyping

For each WISMUT miner, the BfS holds detailed occupational history data, including employment periods, work locations (sites and shafts), job tasks, work areas (underground mining, processing mills, open-cast mining, surface work), and absence periods. Individual radiation exposure (E) were estimated by others retrospectively, linking work tasks, places and durations with annual values for radon and decay products (working level months, WLM), external gamma radiation (mSv), and long-lived radionuclides (kBq·h/m³; LLR) in a Job Exposure Matrix (JEM) [22, 23]. A second JEM covered exposure to fine dust, silica dust, and arsenic, expressed in dust years—defined as 1 mg/m³ (fine dust/silica dust) or 1 µg/m³ (arsenic) over 220 shifts of 8 h each.

### Statistical testing for genetic relative risk

As G-Model we considered the distribution of genotypes $$\:\left[\begin{array}{ccc}{g}_{0}&\:{g}_{1}&\:{g}_{2}\end{array}\right]\:$$among “cases”, taking into account the minor allele frequency f (MAF), the genetic relative risk (RR, risk change per rare allele), with F allowing a deviation from g1 (excess heterozygosity). The distribution of genotypes in the source population is assumed to follow the HWE. Therefore, this is an HWE distortion test. Details are explained in the Additional file 1.

The genetic relative risk for lung cancer (RR) was estimated, independently of any occupational exposure, by mapping the posterior distribution of parameters of the G-model (likelihood x prior distributions of the parameters) within the Bayesian MCMC framework. The mean point estimate and the *95%-Highest Posterior Density Intervals* (95%-HDI) were derived from the a-posterior distributions. A parameter was labelled as significant in this report if $\:2x\:1-{p}_{direct}\:$) (“probability of direction”) was lower than 0.05 [24].

### Statistical testing for gene-environment interaction

For testing gene-environment interaction (GxE), the parameter RR of the HWE distortion test was considered linearly dependent on occupational exposure (now labelled as GxE-model):

$$\:RR={e}^{{\alpha\:}_{RR}+{\beta\:}_{RR}\mathrm{*}{E}^{\tau\:}}$$

Exponentiating the linear relationship ensures that the RR does not take on negative values. The parameter τ enables a data-driven scale transformation of the exposure during the model estimation. If the parameter $\:{\beta\:}_{RR}\:$deviates from zero, an interaction between genotype and exposure can be inferred. The genetic RR is then modified by the exposure of interest. $\:{e}^{{\alpha\:}_{RR}}$ is equal to genetic relative risk RR from the G-Model in the case of no GxE-interaction ($\:{\beta\:}_{RR}=0$). Otherwise $\:{e}^{{\alpha\:}_{RR}}$ is equal to genetic relative risk RR of unexposed individuals (* E=0*; given the linearity assumption is correct).

## Results

### Description of the case series

All lung cancer biospecimens examined here were derived from formalin-fixed, paraffin-embedded (FFPE) lung tissue material from the pathological archive of the former Institute of Pathology of the Health Service of WISMUT SDAG [25, 26], the world’s fourth largest producer of uranium between 1946 and 1990. Biospecimens and phenotypes are stored today in the “German Uranium Miners’ Biobank” (GUMB) [14].

The sample analysed consisted of 611 male miners. They were born between 1899 and 1951 (see Table 2), started mining between 1946 and 1976 when they were between 15 and 50 years old (in average 28 yrs.). Almost all of them (with the exception of four miners) were born before the Second World War (1939), and therefore survived it. 34% were children when the war broke out, 21% were teenagers and 45% were adults. They were employed by the WISMUT Company half of them from the company’s early years, 1946–1949, the others later) for an average of 13 years and lived for a further average of 25 years (up to 41) afterwards. They were exposed to specific hazardous substances of uranium mining between ½ and 41 years. Given this background, whilst this sample represents a unique source to study GxE, it is not representative of the general population. For example, the age at death of the 611 miners was between 37 and 88 years, on average 66 years (median 65 years). This is remarkably low.

This case series is not related to the cohort of WISMUT miners. It includes miners with available biospecimens and data, and is only a small subgroup of all lung cancer cases which occurred among former WISMUT miners. Histological samples were taken from these miners and stored in the company’s own histopathological archive, if there was a selection is unknown – it certainly did not happen by chance – the sample is also not representative nor random of all miners ever employed by WISMUT AG. A further selection is that FFPE tissue blocks from male workers who died from 1986 onwards of lung cancer were chosen to extract DNA as described in detail in Gomolka et al. (2022).

About one in three (36%) had also been diagnosed with silicosis. Adenocarcinomas LUAD, (38%), small cell carcinomas SCLC, (31%) and squamous cell carcinomas SqCLC, (28%) were the most common subtypes.

**Fig. 1:**
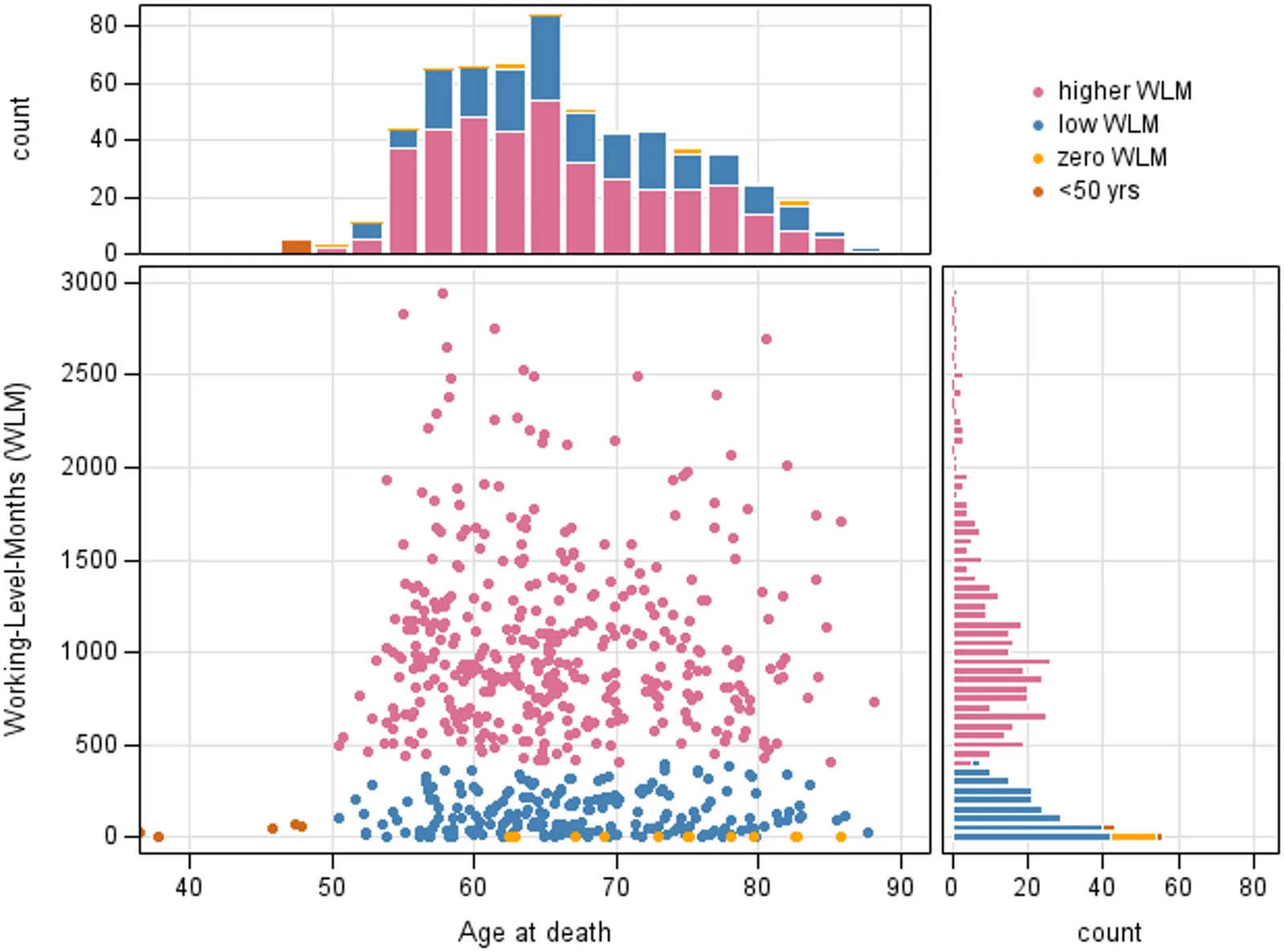
cumulative exposure to radon and its decay products [WLM] by age at death

Cumulative exposure to radon and its decay products, measured in Working-Level-Months (WLM), is considered as the primary exposure. The level of exposure ranged between 0 and almost 3000 WLM, with a median value of 663 WLM see Table 3. However, the WLMs followed a bi-modal left-skewed distribution. One peak is observed at a very low level of exposure (< 50), another peak was observed at about 800 WLM. Around 25% of miners have accumulated more than 1000 WLM during their employment. This distribution was found independent of the age at death (see Table 3; Fig. 1) as of other lung cancer or mining related features. The distribution of other occupational exposures is described in Table 3. These moderately to strongly correlate with each other: weakest - arsenic vs. silica dust (ρ = 0.47) and strongest - LLR vs. gamma radiation (ρ = 0.95). Age at death and year of birth are generally uncorrelated with these mining-specific phenotypes.

No genotypes could be derived for *n* = 85 DNA samples from WISMUT miners (14% of 611). Therefore, data from 526 WISMUT miners were included in the statistical modelling.

**Table 2:** Description of the case series of former WISMUT miners [1]

Category		cumulative radon exposure							
		All		> 50 WLM		< 50 WLM		0 WLM	
		*N*	%	*N*	%	*N*	%	*N*	%
All		611	100%	524	100%	74	100%	13	100%
Smoking [y/n]	**never smoker**		3%	17	3%	2	3%		
	**ever smoker**	128	21%	120	23%	7	9%	1	8%
	**unknown**	464	76%	387	74%	65	88%	12	92%
Silicosis [y/n]	**no**	392	64%	311	59%	68	92%	13	100%
	**yes**	219	36%	213	41%	6	8%	.	.
Histology	**LUAD**	231	38%	193	37%	35	47%	3	23%
	**SCLC**	189	31%	162	31%	21	28%	6	46%
	**SqCLC**	170	28%	149	28%	18	24%	3	23%
	**others**	20	3%	19	4%			1	8%
	**LCLC**	1	< 1%	1	< 1%				

ever smoker: miners who have reported smoking at least once during their period of employment;

LUAD Adenocarcinomas, SCLC Small cell carcinoma, SqCLC Squamous cell carcinoma, LCLC large cell carcinomas

**Table 3:** Description of the occupational exposure

	*N*	Minimum	25% Percentile	50% Percentile	Mean	75% Percentile	Maximum	s.d.
cumulative exposure to …								
radon and its decay products [WLM]	611	0	177.8	663.1	727.2	1083.2	2947.4	590.4
LLR [kBq·h/m³]	611	0	1.3	4.4	8.8	12.2	81.4	10.9
gamma radiation[mSv]	611	0	8.3	32.7	71.9	96.7	576	92
fine dust [mg/m³·Jahre]	611	2.4	46.9	82.8	86.1	118.4	238.8	48.8
silica-dust [mg/m³·years]	611	0.4	8.2	15.1	15.8	22.1	51.4	9.2
arsenic [µg/m³·years]	596	0	0	31.9	95.1	126	1132.9	149.7

WLM Working-Level-Months, with weighting, s.d. standard deviation; LLR long-lived radionuclides

### G model: genetic relative risk

**Fig. 2:**
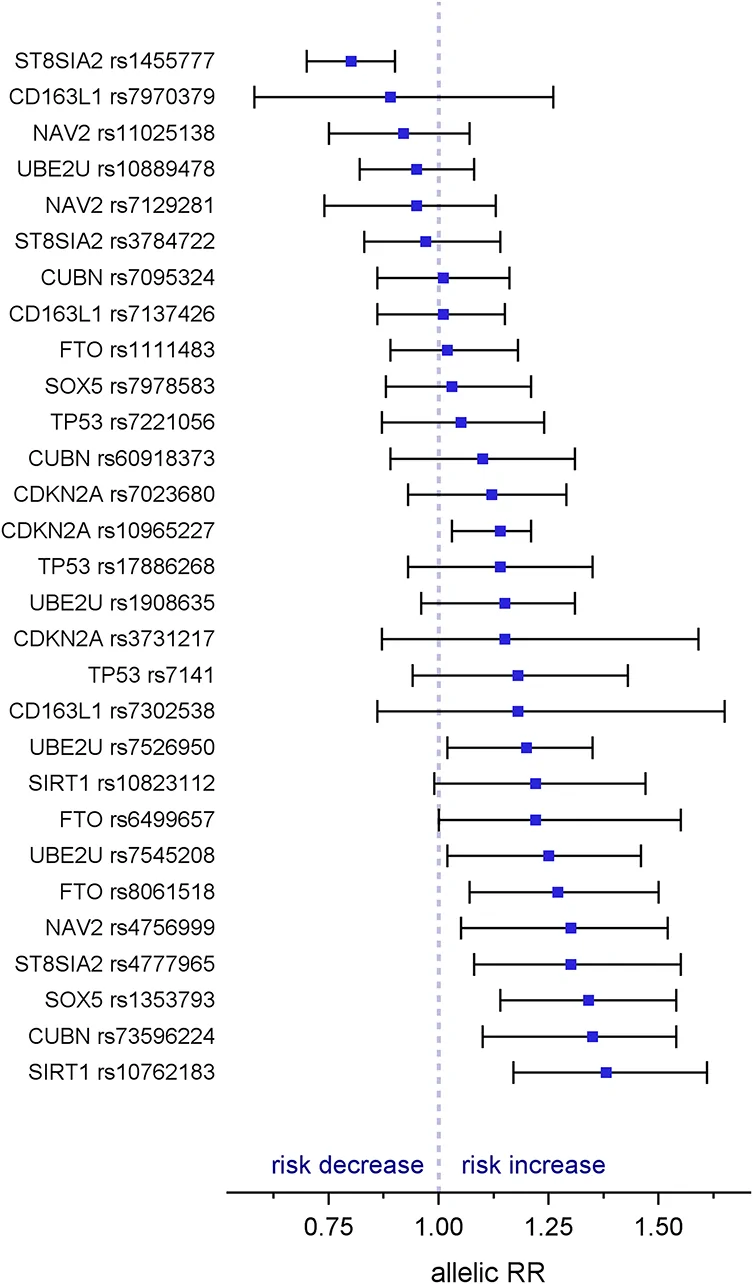
Genetic relative risk estimated by the G-model). RR: genetic relative risk per rare allele

The genetic relative risks RR risk change per rare allele), estimated by fitting the G-model, range from 0.80 (rs1455777-*ST8SIA2*) to 1.38 (rs10762183-*SIRT1*) (see Fig. 2). A significant increase in lung cancer risk per minor allele was observed for 9 of the 29 markers analysed, and a significant reduction was observed for one marker (*p* < 0.05). Three further markers with similarly high RRs (from the *TP53*, *SIRT1* and *FTO* genes) fell just short of the significance threshold (see S-Table).

The strongest genetic risk increase was observed for the marker rs10762183 (SIRT1) with a genetic RR = 1.38 (95%-HDI 1.17–1.61, *p* < 0.0001), the second strongest genetic risk increase per rare allele for the marker rs1353793 (*SOX5*) with an RR = 1.34 (95%-HDI 1.14–1.54, *p* < 0.0001). A comparable genetic risk increase of RR = 1.35 (95%-HDI 1.10–1.54, *p* < 0.0073) was observed for the marker rs73596224 (CUBN). Other markers with significant RRs can be assigned to the genes *CDKN2A*, *CUBN*, *FTO*, *NAV2*, *ST8SIA2* and *UBE2U*.

### GxE model: interaction for radon exposure [in WLM]

**Fig. 3:**
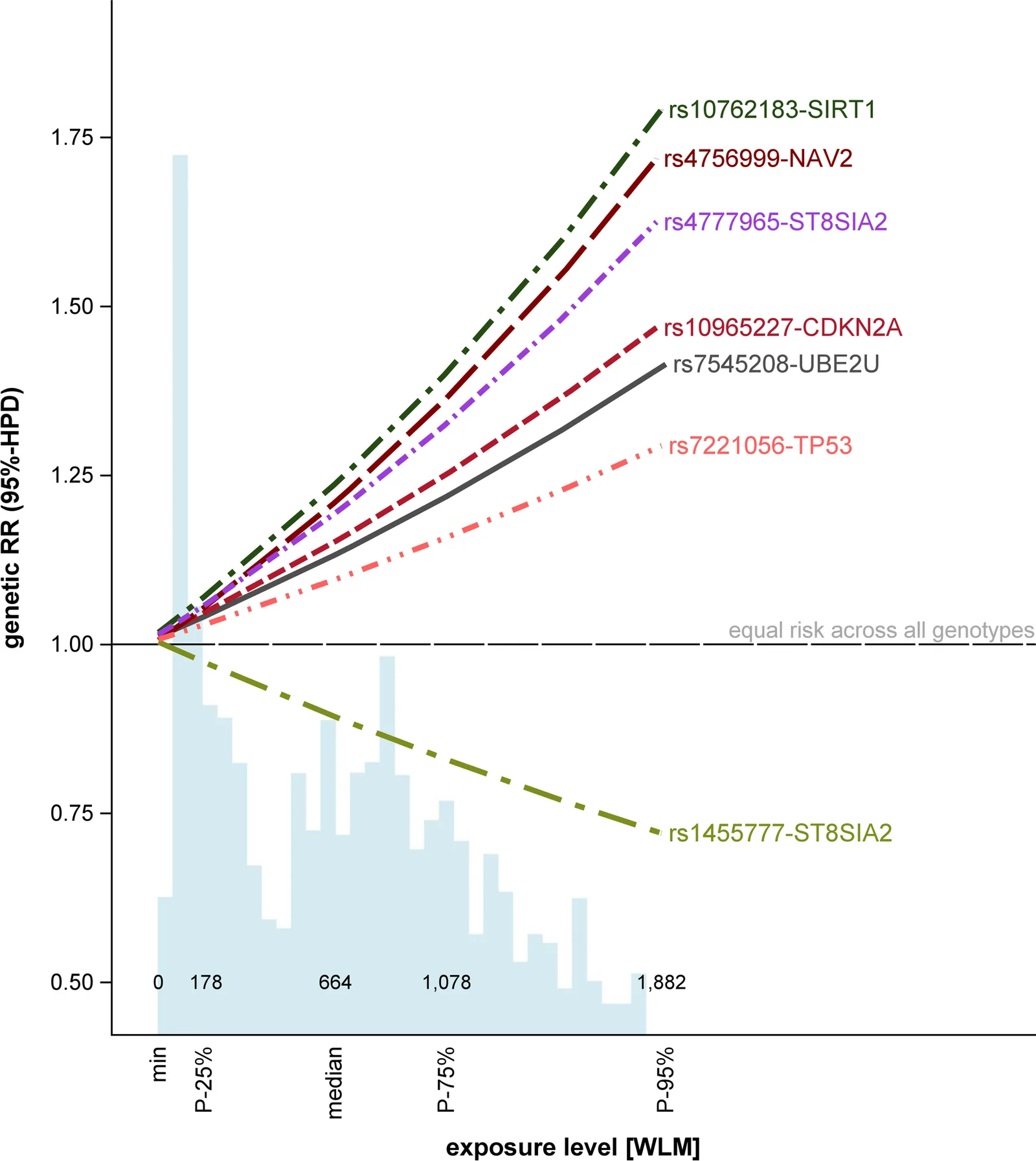
RR genetic relative risk; the distribution of radiation exposures in WLM is shown in pale blue in the background. The minimum and the 25%, 50% and 75% percentiles are highlighted on the x-axis. RR = 1 all genotypes have the same genetic RR. Horizontal curve gradients would indicate that the relative genetic risk does NOT change with the degree of exposure and therefore there is no GxE-interaction. In a pure GxE-interaction, the genetic relative risk should be 1 at zero exposure starting point of the line)

A significant modification of the genetic RR by radon exposure [in WLM] was observed for seven of the 29 markers from 6 of the 10 genes analysed (see Fig. 3). The RR increases almost linearly by WLM. For six of these markers a significant genetic relative risk (RR) was found (see above). The 7th marker is rs7221056 (*TP53*). In the model the scale parameter $\:\tau\:$ was estimated as almost 1 for all markers, the exposure was not rescaled in estimating RR.

**Fig. 4:**
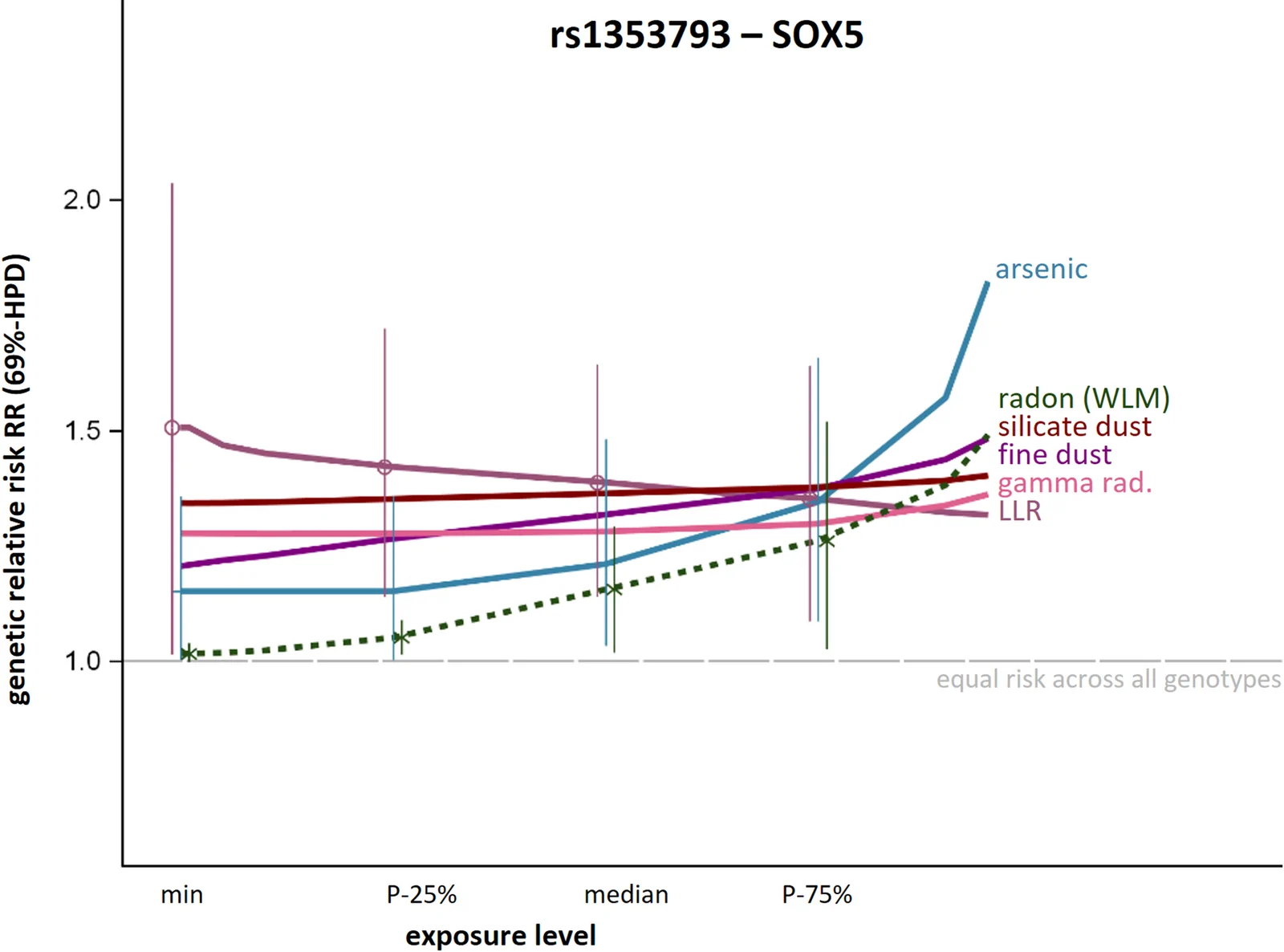
GxE-interaction of rs1353793 SOX5) with all exposures for selected markers. A relative genetic risk of RR = 1 means that all genotypes have the same genetic RR. Horizontal curve gradients would indicate that the relative genetic risk RR does NOT change with the degree of exposure and therefore there is no GxE interaction. In a pure GxE interaction, the genetic relative risk should be 1 at zero exposure (starting point of the line). For visual comparison, the exposures were scaled so that the quartiles are positioned identically. The minimum and the 25%, 50% and 75% percentiles are highlighted on the x-axis. The curves for each exposure were scaled so that the percentiles correspond graphically

For all markers, a genetic RR of almost 1 was estimated at minimal exposure WLM = 0 (see Fig. 3). This means that the risk of lung cancer without being exposed does not depend on the genes investigated. The risk modification therefore appears to result exclusively from the interaction between genes and as modelled here, radon exposure (Fig. 4). This clear picture of “no genetic risk without occupational exposure” did not apply to the other exposures considered.

The two markers with the strongest positive interaction $\:{\beta\:}_{RR}>0$) are rs4756999 (*NAV2*, *p*=0.0002) and rs10762183 (*SIRT1*, *p* = 0.0014). The genetic RRs derived from $\:{\alpha\:}_{RR}$, $\:{\beta\:}_{RR}$ and $\:\tau\:$ at low (178 WLM), median (633 WLM) and high (1083 WLM; 75% percentile) exposure levels are about 1.05, 1.21 and 1.36, respectively (see Table 4). We also observed interaction with WLM for the marker rs7545208 *UBE2U*, *p* = 0.0187), but slightly less pronounced RRs at low, median and high exposure: 1.04, 1.13 and 1.22).

Two markers with significant interaction effects (rs1455777 and rs4777965) belong to the gene *ST8SIA2*. While rs4777965 shows an interaction comparable to the above markers, $\:{\beta\:}_{RR}$ is negative for rs1455777. This means that the risk of disease under radon exposure decreases with the number of rare alleles. The derived genetic RRs at low, median and high exposure are estimated to be 0.97 (= 1/1.03), 0.89 (= 1/1.12) and 0.83 (= 1/1.20).

For 4 markers with significant genetic RRs rs7526950- *UBE2U*, rs73596224-*CUBN*, rs1353793-*SOX5* and rs8061518-*FTO*) no interaction with radon exposure (in WLM) could be shown to be significant.

**Table 4:** Estimated modification of RR by radon exposure [in WLM] extract)

	$\:{\boldsymbol{\beta\:}}_{\boldsymbol{R}\boldsymbol{R}}$	*p*-value	0 WLM		178 WLM		663 WLM		1083 WLM	
			RR	95%-HDI	RR	95%-HDI	RR	95%-HDI	RR	95%-HDI
rs4756999 - *NAV2*	0.00030	0.0002	1.01	0.99–1.03	1.05	1.02–1.09	1.21	1.08–1.35	1.36	1.12–1.63
rs1455777 - *ST8SIA2*	-0.00018	0.0003	1.00	1.00-1.01	0.97	0.96–0.99	0.89	0.86–0.93	0.83	0.78–0.89
rs4777965 - *ST8SIA2*	0.00021	0.0046	1.01	1.00-1.04	1.06	1.02–1.10	1.19	1.06–1.34	1.32	1.09–1.57
rs10762183 - *SIRT1*	0.00024	0.0014	1.02	0.99–1.05	1.07	1.02–1.12	1.24	1.10–1.38	1.40	1.15–1.67
rs7545208 - *UBE2U*	0.00015	0.0187	1.01	0.99–1.03	1.04	1.01–1.08	1.13	1.01–1.26	1.22	1.00-1.45
rs7526950 - *UBE2U*	0.00008	0.3849	1.01	0.99–1.03	1.03	0.98–1.07	1.07	0.94–1.19	1.11	0.90–1.32
rs1353793 - *SOX5*	0.00017	0.0813	1.02	1.00-1.04	1.05	1.01–1.09	1.16	1.02–1.29	1.26	1.03–1.52
rs73596224 - *CUBN*	0.00017	0.2234	1.00	0.97–1.03	1.04	0.98–1.09	1.14	0.94–1.34	1.24	0.88–1.60
rs8061518 - *FTO*	0.00003	0.7404	1.02	1.00-1.04	1.03	1.00-1.07	1.07	0.95–1.20	1.11	0.91–1.33

RR genetic relative risk estimated from the GxE model; 95%-HDI 95% Highest Posterior Density Interval 178, 663 and 1083 WLM are the 25%, 50% and 75% percentile of the exposure in the collective the table is sorted by p_directly_ in ascending order, but markers of the same gene are kept together

### GxE model: interaction for other exposures

Other than for radon exposure [in WLM], only four (out of the 29 × 5 = 145; 3%) investigated marker-exposure interactions were statistically significant (*p* < 5%). Two of these significant GxE interactions were observed in relation to arsenic, one each in relation to LLR or gamma radiation, but none in relation to fine dust or silica dust.

The scale parameter τ was estimated as almost 1 for radon and arsenic exposure for most markers. With regard to LLR radiation, τ ranged from 0.3 to 0.6, and with regard to gamma radiation, from 0.70 to 0.98, and with regard to fine dust or silica dust, it averaged 0.40 and 0.45, respectively. This underlines the importance of scale-adapted modelling for each exposure.

As can be seen in Fig. 4; Table 5, a GxE interaction of marker rs1353793 (SOX5) can be observed for radon almost *p* = 0.0813) and for arsenic statistically significant (*p* = 0.0231), but not with any of the other exposure variables (horizontal lines indicate no GxE interaction). This, together with the correlation between the two exposure variables (*r* = 0.48), makes it difficult to regard either of them as the cause of the observed interaction. No significant interaction was observed for the other two markers of the *SOX5* gene. The situation, however, is similar with regard to the marker rs4777965 and rs1455777, whose interaction with arsenic (*p* = 0.0144; 0.0551) and with radon (*p* = 0.0046; *p* = 0.0003) was, statistically speaking, below or on the threshold of significance (Table 5 and S-Figure 1 in the Additional file 1).

**Fig. 5:**
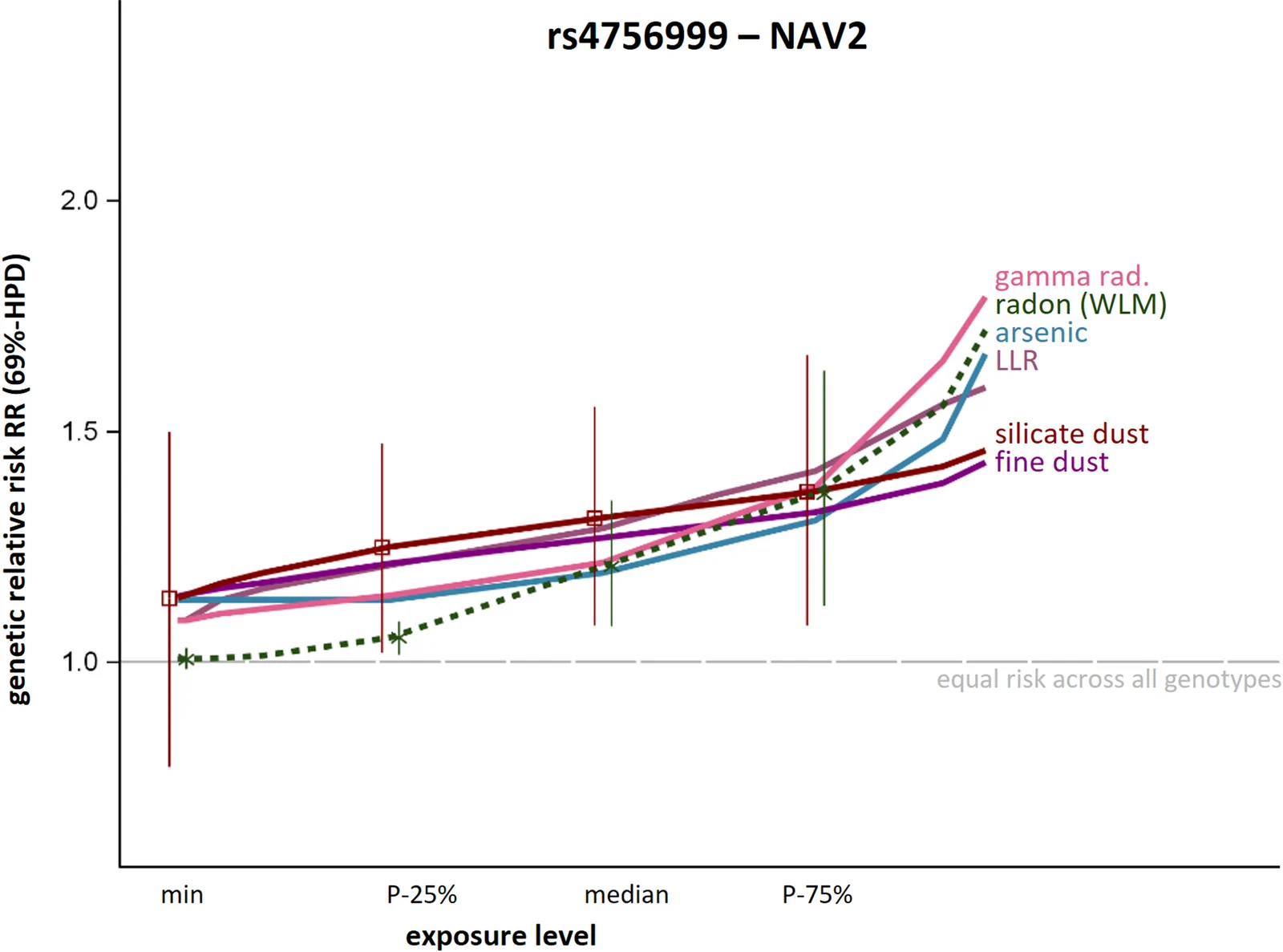
GxE-interaction of rs4756999 NAV2) with all exposures for selected markers. A relative genetic risk of RR = 1 means that all genotypes have the same genetic RR. Horizontal curve gradients would indicate that the relative genetic risk RR does NOT change with the degree of exposure and therefore there is no GxE interaction. In a pure GxE interaction, the genetic relative risk should be 1 at zero exposure (starting point of the line). For visual comparison, the exposures were scaled so that the quartiles are positioned identically. The minimum and the 25%, 50% and 75% percentiles are highlighted on the x-axis. The curves for each exposure were scaled so that the percentiles correspond graphically

**Fig. 6:**
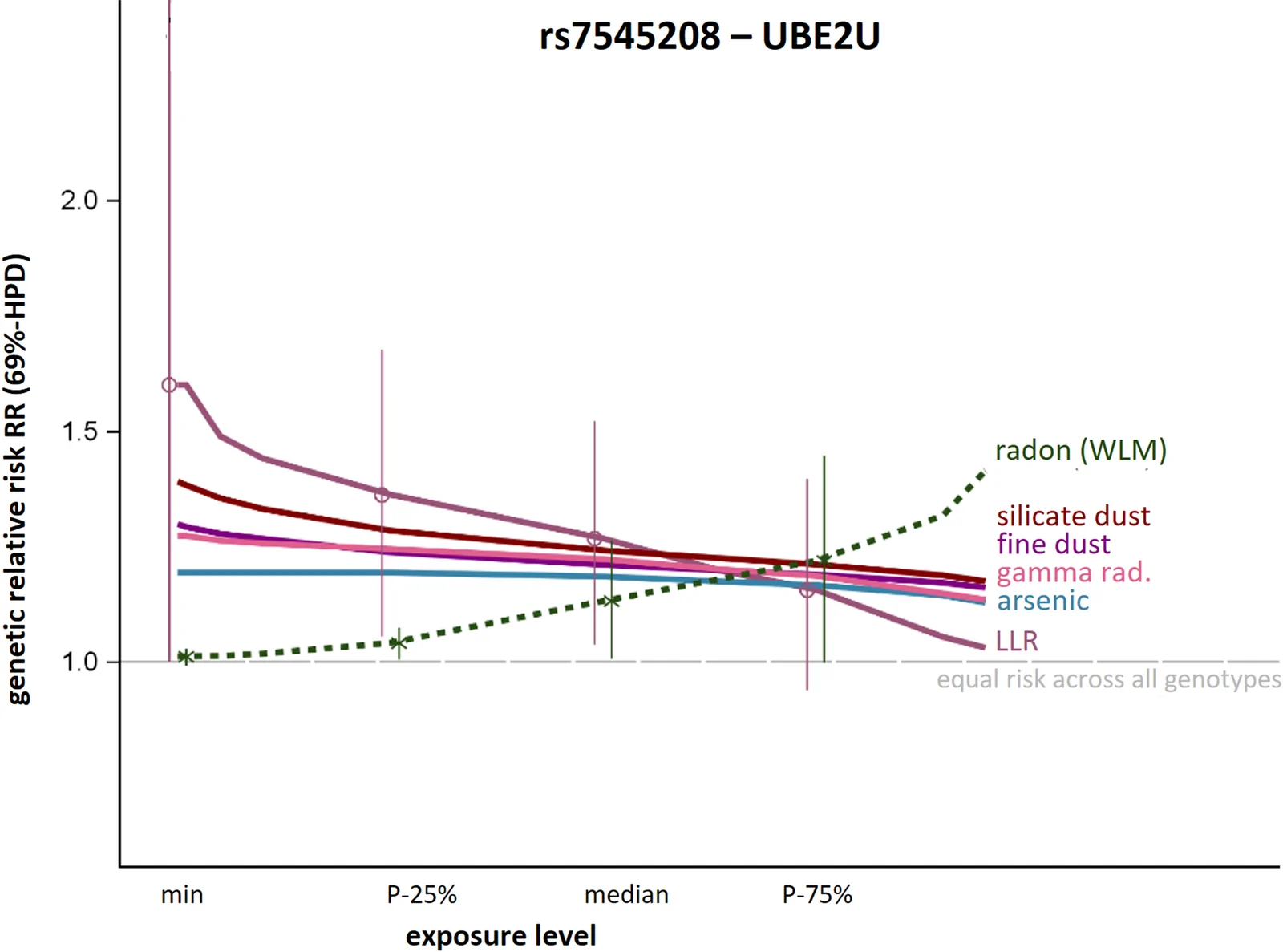
GxE-interaction of rs7545208 UBE2U) with all exposures for selected markers. A relative genetic risk of RR = 1 means that all genotypes have the same genetic RR. Horizontal curve gradients would indicate that the relative genetic risk RR does NOT change with the degree of exposure and therefore there is no GxE interaction. In a pure GxE interaction, the genetic relative risk should be 1 at zero exposure (starting point of the line). For visual comparison, the exposures were scaled so that the quartiles are positioned identically. The minimum and the 25%, 50% and 75% percentiles are highlighted on the x-axis. The curves for each exposure were scaled so that the percentiles correspond graphically

Although only significant for radon (*p* = 0.0002), a GxE interaction of the marker rs4756999 (*NAV2*; Fig. 5) with other exposure variables (LLR and gamma radiation, fine dust, silica dust and arsenic) cannot be ruled out. The same appears for the markers rs10762183 (*SIRT1*), rs7221056 *(TP53*), and rs10965227 (Table 5 and S-Figure 1 in the Additional file 1) with a significant interaction with radon (*p*_*SIRT1*_=0.0014, *p*_*TP53*_=0.0303, *p*_*CDKN2A*_=0.0252), however, no interaction to arsenic all (p > 0.1). Solely marker rs7545208 (UBE2U; Fig. 6) shows no GxE interaction other than with radon (*p* = 0.0187; when τ is set to 1).

**Table 5:** Significance of GxE interaction by exposure

Marker	Gene	radon and its decay products [WLM]	LLR [kBq·h/m³]	gamma radiation [mSv]	arsenic [µg/m³·years]	particulate matter [mg/m³·Jahre]	silica-dust [mg/m³·years]
rs7545208	UBE2U	**0.0187**	0.2697	0.4950	0.7034	0.6328	0.5785
rs7526950	UBE2U	0.3849	0.6903	0.4132	0.2284	0.8736	0.3191
rs10889478	UBE2U	0.6923	0.6918	0.3209	0.8911	0.5588	0.5625
rs1908635	UBE2U	0.6855	0.4752	0.8365	0.8360	0.3407	0.8203
rs7023680	CDKN2A	0.1515	0.0980	0.7233	0.4868	0.9287	0.6871
rs3731217	CDKN2A	0.3578	0.4368	0.3262	0.7665	0.2441	0.2320
rs10965227	CDKN2A	**0.0252**	0.4182	0.3781	0.0897	0.5800	0.3674
rs73596224	CUBN	0.2234	0.2804	0.2838	0.7519	0.5414	0.6404
rs60918373	CUBN	0.4681	0.8135	0.7697	0.6709	0.3753	0.3769
rs7095324	CUBN	0.4638	0.6074	0.7711	0.8652	0.4019	0.4736
rs10823112	SIRT1	0.1244	0.9675	0.6893	0.2862	0.1171	0.2268
rs10762183	SIRT1	**0.0014**	0.1031	0.2757	0.1455	0.2896	0.0955
rs7129281	NAV2	0.9352	0.2789	0.7028	0.8333	0.5258	0.1691
rs11025138	NAV2	0.3065	0.2973	0.1198	0.4753	0.3706	0.6067
rs4756999	NAV2	**0.0002**	0.1085	0.0712	0.8659	0.3298	0.4740
rs7137426	CD163L1	0.6735	0.1914	0.2736	0.8369	0.8089	0.9262
rs7970379	CD163L1	0.7757	0.6644	0.4348	0.8902	0.8329	0.3626
rs7302538	CD163L1/ACSM4	0.5323	0.4243	0.5861	0.3549	0.5892	0.6776
rs1353793	SOX5	0.0813	0.6878	0.4794	**0.0231**	0.8948	0.7339
rs7978583	SOX5	0.1811	0.6553	0.3021	0.7425	0.7732	0.6004
rs4777965	ST8SIA2	**0.0046**	0.2791	0.3009	**0.0144**	0.4123	0.2811
rs1455777	ST8SIA2	**0.0003**	0.2940	0.3742	0.0551	0.4457	0.0618
rs3784722	ST8SIA2	0.3993	**0.0191**	**0.0005**	0.0850	0.2331	0.1816
rs8061518	FTO	0.7404	0.3895	0.4751	0.3596	0.6355	0.3778
rs6499657	FTO	0.7741	0.6435	0.6587	0.3145	0.9741	0.9904
rs1111483	FTO	0.7604	0.6355	0.3752	0.4556	0.6169	0.6915
rs17886268	TP53	0.9532	0.5852	0.8059	0.8986	0.8802	0.4611
rs7141	TP53	0.1025	0.1052	0.0723	0.0907	0.2683	0.6897
rs7221056	TP53	**0.0303**	0.1427	0.2613	0.2761	0.3669	0.3049

^ŧ^ for rs7545208 a p-value of **0.0187** was observed considering WLM, when fixing τ at 1

## Discussion

Our aim was to validate and replicate previously reported *interactions of radon with genomic loci* (radon-gene interactions)* with respect to lung cancer risk* in an independent series of 611 DNA samples from uranium miners, all former employees of WISMUT AG in Germany. *T*his sample represents a unique source to study for GxE, however it is neither representative of the general population nor for all miners ever employed by WISMUT AG. E.g. the proportions of LUAD and SqCLC correspond to the proportions among all lung cancer cases in Western Europe in 2020 (39% and 31%, respectively), while the proportion of SCLC is higher than in Western Europe (15%). Only one WISMUT miner (< 1%) was diagnosed with large cell carcinoma (LCLC; Western Europe: 7%) [27].

In reviewing the literature, we extracted 34 genes modifying the Radon-induced lung cancer risk.

The histological samples for studying genetic interactions with occupational radon exposure had been archived for a long time and recently isolated DNA from normal formalin-fixed paraffin-embedded lung tissue samples were therefore highly fragmented due to specific fixation procedures at that time. Despite the poor quality of the DNA, after setting up a robust NGS analysis method based on specifically designed short amplicons (< 140 bp) to type degraded genomic DNA and optimization of the procedure we were able to genotype 29 markers from 10 of the 34 literature-reported genes (*FTO*, *UBE2U*, *CUBN*, *ST8SIA2*, *SOX5*, *TP53*, *CD163L1*, *CDKN2A*, *NAV2*, and *SIRT1*).

Genotypes could be determined for the first time in this material by NGS in 526 of the 611 samples. Because NGS results could often not categorized into 0, 1 or 2 minor alleles, a reliable translation of NGS results (genotype doses) into genotypes (or genotype probabilities) has proven to be a methodological challenge. Therefore, we first developed a flexible mathematical function for the isotonic conversion of doses into probabilities (FLDSf), specifically for this purpose [21]. Subsequently, the genotype probabilities for each marker were estimated based on NGS reads, taking into account various NGS settings. We found that it was necessary to apply 12 different translations to the NGS reads to reliably fit statistical models to the uncertainties of the genetic data. When integrated into statistical models, we were able to detect significant modification in genetic RR due to radiation exposure [in WLM] for seven of the 29 markers from six of the ten genes examined.

Statistical significance, even in a replication study like this, should not be the only justification in postulating the existence of GxE interaction. The evidence for significant interactions between different genes and environmental exposures derived from the data is only corroborated by proven biological functional relationships within carcinogenesis or any known associations with lung cancer risk.

Markers in three genes show a clear dose-dependent modulating effect only in interaction with radon, *UBE2U*, an enzyme responsible for ubiquitination of proteins, *SIRT1*, a central epigenetic regulator and a genetic marker located in the *CDKN2A/CDKN2B/CDKN2B-AS1* gene cluster.

Genetic markers in the gene *UBE2U*, that modulate the risk for radon-induced lung cancer, were first discovered in our preceding genome-wide interaction study in Uranium miners embedded in a broader case control study of 15,077 cases and 13,522 controls of European ancestries [10]. In the present study, the significant interaction with radon could be confirmed for the marker rs7545208, one of three investigated markers assigned to *UBE2U*. Ubiquitination affects proteins and important pathways in many ways. Pairing of E2-E3 enzymes regulating ubiquitination have been implicated in cancer initiation and progression or cancer suppression when cancer suppressors are degraded [28]. *UBE2U* has been shown to have a central regulatory function to regulate tumor suppressor proteins involved in DNA double-strand break repair, cell cycle progression and checkpoint control. The *UBE2U* gene is known to influence the response to DNA damage and other important proteins in double-strand break repair, e.g., via regulation of 53BP1. *UBE2U* interacts with the E3 ligase RNF17 to enforce DNA damage responses by promoting the accumulation of 53BP1 at ionizing radiation-induced double-strand breaks DSBs) [29]. Thus, mutations in the *UBE2U* gene may tip the balance toward genomic instability and oncogenic progression. Further studies are needed to elucidate the exact mechanism in lung cancer.

*SIRT1* was the first gene identified predisposing for squamous cell carcinoma in the lung in interaction with radon in a cohort of former uranium miners from the Colorado Plateau [12]. A significant association between *SIRT1* gene polymorphisms and human NSCLC was neither found in a Chinese study without radon exposure, nor in a GWAS on lung cancer or other lung diseases [30]. However, the Chinese study was rather small and an increased OR of 1.7 was observed but did not reach significance. In our study, the interaction observed for the marker rs10762183 (SIRT1) is also likely to be attributable to the radiation component, confirming data from the US study. As a hub gene in cancer-related pathways, *SIRT1* plays a central role as an epigenetic regulator in a variety of cellular signalling mechanisms and triggers cell defence and cell survival in the event of radiation-induced damage [31, 32]. Nonetheless, many aspects of the *SIRT1*–cancer relationship are ambiguous. However, *SIRT1* contributes to DNA repair after irradiation [12] as component of double-strand break (DSB) repair through homologous recombination (HR), non-homologous end joining (NHEJ), DNA damage response (DDR) and nucleotide excision repair (NER) [33]. Although it is not yet clear whether *SIRT1* acts as a tumour promoter or tumour suppressor in relation to cancer in general [34], radon is assumed to be the interacting exposure.

A gene cluster at chromosome 9p21 encodes three tumour suppressor genes - *CDKN2A*, CDKN2B and the antisense non-coding RNA CDKN2B-AS1 (also known as ANRIL), and further MTAP, a gene encoding an enzyme for the salvage of both adenine and methionine. They are frequently mutated and co-deleted in a wide variety of tumours, including lung cancer [35]. Two different markers located in CDKN2B-AS1 were linked to lung cancer in individuals of European decent. The SNP rs1333040 showed, amongst others, a genome-wide significant association with SqCLC [36]. rs62560775 showed a suggestive significant association with LUAD [37]. In addition, the SNP rs885518, located in MTAP, was found in the first node of a decision tree of genomic markers to identify lung cancer cases in never smokers [38]. *CDKN2A* and − 2B are inhibitors of CDK4 kinase. Both genes are considered tumour suppressor genes with a central role in cell cycle regulation, including in lung cancer [37]. The gene product of CDKN2B-AS1 is a functional RNA molecule and is, e.g., involved in the regulation of *CDKN2A* and − 2B [35]. *CDKN2A* was recently labelled as hub gene for LUAD [39]. With regard to occupational radiation exposure, epigenetic dysregulation of *CDKN2A* was observed in nuclear power plant workers in Mayak, Russia, who were exposed to alpha radiation [13]. *CDKN2A* gene expression is regulated by methylation, and methylation rates have been reported to be affected by radon exposure in Chinese uranium miners [40]. The GxE interaction observed for the marker rs10965227, located close to rs1333040 within CDKN2B-AS1, was most significant for radon, but its magnitude is comparable with that of other exposures. However, there is only little to moderate LD between the four mentioned markers D’: 0.003–0.742, European populations [41]). We therefore see strong evidence that this gene complex is a complex predictor of lung cancer, partly due to interactions with radon.

### Gene-environment interaction due to arsenic occupational exposuree

*ST8SIA2* was first identified to influence lung cancer risk in interaction with radon in our previous GWAS study. *ST8SIA2* expression is linked to NSCLC recurrence risk and SCLC prognosis [42, 43], and its genomic markers associate with respiratory impairment (airflow obstruction [44]) and smoking cessation in Asians [45]. Here, we observed an interaction not only due to radon exposure but also with other occupational exposure variables of the miners. Based on the pathway described below, we believe that the interaction with rs4777965 should be attributed to the risk of lung cancer from arsenic exposure rather than radiation exposure. Arsenic is classified as a Class 1 human carcinogen by the International Agency for Research on Cancer with strong evidence for lung cancer [46, 47]. The observed interaction is in our study supported by recent functional data of *ST8SIA2*. *ST8SIA2* is a polysialyltransferase that synthesizes polysialic acid (PSA) on substrates like the neural cell adhesion molecule NCAM, enhancing tumour cell migration. It may promote tumorigenesis by shielding tumour cells from immune detection via a protective sialic acid layer. It has recently been discovered that arsenite increases the sialic acid content on the cell surface by inhibiting sialidase activity [48].

The same applies to *SOX5*, a transcription factor of the high mobility group box (SOX) family. It has been more strongly associated to arsenic exposure rather than radon exposure. We observed a G×E interaction with arsenic exposure at marker rs1353793 of *SOX5*. A link between arsenic exposure and tumour induction has not yet been described, although *SOX5* has been implicated in accelerating migration and invasion via epithelial-mesenchymal transition. *SOX5* has been described as an oncogene in many types of cancers, including lung cancer [49]. However, four other markers of *SOX5* (rs1391789, rs12367235, rs1259887, rs1800973) have been associated with C-reactive protein (CRP) levels and are partly in strong LD with rs1353793 [50]. CRP is considered a non-specific biomarker of acute inflammation. Arsenic, on the other hand, can alter proliferation status and expression of key pro-inflammatory cytokines (e.g. TNF-α, IL-1, IL-6). It has been proven that exposure to arsenic can trigger an inflammatory response and that lungs are particularly susceptible to arsenic [51–53]. In summary, the observed interaction of *SOX5* markers with arsenic could be caused by genomic linkage to CRP [54].

Further observed GxE interactions should be viewed critically, although their significance has been reiterated.

We found *NAV2*, a member of the neuron navigator family, for the first time as a possible susceptibility gene modulating the radon-induced lung cancer risk. Our study also confirmed this previously found interaction. However, it was observed that the interaction was comparable in strength to the other exposures considered. *NAV2* is considered as a pro-tumorigenic factor for colorectal cancer. The Human Protein Atlas [18] identifies *NAV2* as a “multiplayer” due to a high “protein expression score” in many tissues/organs (especially the heart and lungs). Yet, little is known about possible functional pathways of members of the neuron navigator gene family. They may play a role in cell migration and tumour invasion [55, 56]. The functional evidence to support the observed GxE interaction needs clearly to be further elaborated to strengthen the putative *NAV2* radon-induced lung cancer risk.

*TP53* is a central hub gene in cancer progression, with almost half of all human tumours harbouring a mutation in this gene. The coded protein acts as multifunctional tumour suppressor. Mutations in the *TP53* gene are the most common somatic changes in human cancer tissue that occur during carcinogenesis [57]. Numerous rare mutations in the TP53 gene have been identified in tumour tissue from miners and smokers, with mutations at codon 249 being more common in both these groups [58–60]. Germline variants of this *TP53* codon are classified pathogenic for e.g. the Li Fraumeni Syndrome or the Hereditary Cancer-Predisposing Syndrome [61]. These people affected are at risk of developing many types of cancer and are sensitive to radiation. However, all these is relate to tumour tissue and therefore more closely associated with tumour initiation or development than with susceptibility. TP53 Arg72Pro (rs1042522, located in codon 72) is the only genomic marker for which an elevated heterozygosity was observed in cases of radon-induced LC [62]. For the same marker, a significant association with LC was observed amongst Asians [63, 64] and amongst African Americans, but not amongst European descendants [65, 66]. Because all three genomic polymorphisms of codon 249 are almost monomorphic in Europeans [67] and the reported codon 72 was not association within Europeans we decided to investigate other variants of this *TP53* region. Evidence for GxE in miners for the investigated markers is provided by the upper mentioned GWAS, foremost for rs7221056. 10) Only for this marker we found evidence for GxE in this replication. Yet, this cannot be definitively attributed to radon. No evidence for GxE is provided for rs7141, a weak proxy SNP for the *TP53*Arg72Pro mutation (rs1042522). It therefore stands to reason that, whilst *TP53* may play a role in the development of radon-induced lung tumours, it is of little significance in terms of susceptibility to them.

Apart from the GxE interactions replicated, we proved NGS to be an adequate technique for typing historical, highly fragmented DNA samples. However, due to uncertainties in genotyping results, adequate translation of genotype doses into genotype probabilities was necessary to account for this uncertainty. This was achieved by implementing a sophisticated model before performing data analysis.

Although the smoking status of the miners studied (all of whom were male) is largely unknown, it can be assumed by analogy with the general population that 9 out of 10 probably smoked [68]. This therefore does not raise the question of whether the observed effects should be interpreted as mixed effects resulting from a smoking-radon interaction amongst highly exposed miners. However, it should be critically considered whether the observed gene-radon interactions are in fact attributable to a gene-smoking interaction. None of the genes investigated, or their genomic markers, are known to interact with smoking in relation to lung cancer. However, theoretical links – though not directly substantiated – may be suspected.

For example, *CDKN2A* hyper-methylation was significantly associated with cigarette smoking in tumour tissues of Japanese, Chinese and American lung cancer cases [69]. 

Secondly, it is thought that *SIRT1* (as well as other genes in the SIRT family) interacts with oxidative stress and DNA repair mechanisms, both known to be introduce by tobacco smoke [70, 71]. However, it is not yet clear whether *SIRT1* acts as a tumour promoter or a tumour suppressor in relation to cancer in general [34, 72].

And thirdly, tobacco constituents in the body disrupt the ubiquitin-proteasome system and alter the ubiquitination of many proteins. This can, for example, lead to impaired DNA damage repair and thereby promote the development of lung cancer. The ubiquitination of a protein is mediated by a sequential enzymatic reaction involving E1 activation) – E2 conjugation) – E3 ligase), with *UBE2U* encoding one of the many E2 enzymes. It is worth noting that *UBE2N* was associated with lung cancer in people of European descent [73].

However, our aim is to replicate previously reported GxE effects, most of which have been adjusted for the influence of smoking. And even if such as yet unproven gene-tobacco-lung cancer risk pathways exist, it is questionable if this explains the observed gene-radon-lung cancer interactions. Radon like other occupational exposures) was modelled continuously in the GxE analysis. It is extremely unlikely that, for example, radon exposure would correlate strongly with tobacco exposure, which would be a necessary prerequisite for the alternative explanation. That would only be the case if work assignments had been planned according to individual cigarette consumption. Please note: Kreuzer et al. 2008) report on the WISMUT cohort that *“the low correlation between smoking and cumulative radon exposure makes smoking an unlikely major confounder*.*”* [74].

### Limitations

The histological samples of normal tissue examined had been stored for a long period in the pathological archive. This resulted in isolation of highly degraded DNA from FFPE which were difficult to type even by the carefully designed robust NGS method applied. The uncertainty of the genotype results needed therefore to be specifically modelled, whilst this was highly successful, it still bears a degree of uncertainty. As non-buffered formalin was used at the time of preservation, the isolated DNA quantity and quality limited strongly the number of markers amenable to genotyping.

The restricted sample size and the missing of cancer-free control subjects were determined by the circumstances to work with long-term archived material than by considerations of study design and statistical power. A fixed, limited sample size and the absence of cancer-free control subjects are further implications of the analysis of these histological samples. We are aware of the constraints of a pure case-only design and the limited statistical power [75, 76]. It was therefore necessary to narrow the investigation down to candidate markers which, for example, had been identified in our earlier GWA study of uranium mine workers. We are aware that also other genes especially involved in DNA repair processes and in relation to indoor radon concentrations may also modulate radon-induced lung cancer risk in miners but could not be investigated here [77].

To estimate and test for GxE interaction using the case-only design employed here, we need to strictly assume independence between genotype G and environmental factor E [78]. We consider this assumption to be met, as it is unlikely that a miner’s genetic makeup would influence their assignment to a particular workplace (mine, above ground) and therefore exposure group. Distortions of genotype frequency due to admixing of different ethnic groups can be neglected since all 611 miners are of European descent. However, a distortion of the results due to the healthy worker effect or healthy worker survivor bias in the high exposure group cannot be ruled out in this sample of miners [79]. We must also assume that the 14% of genotypes (from 85 miners) that could not be determined do not cause any distortion.

## Conclusion

For markers from six of the 10 genes examined (UBE2U, *CDKN2A*, *SIRT1*, *NAV2*, *ST8SIA2*, *TP53*), a significant modification of the genetic relative risk for lung cancer due to mining-specific radon exposure [in WLM] was replicated/validated. Radon exposure may be the main cause of some of the observed GxE interactions involving *UBE2U*,* CDKN2A* and *SIRT1*, but it may not be the only one. *This* conclusion is based on our findings and is supported by the functional relevance of the genes, as extracted from the literature. For another marker of gene *SOX5*, a GxE interaction can presumably be attributed to exposure to arsenic.

## Supplementary Information

Below is the link to the electronic supplementary material.

Supplementary Material 1

## Data Availability

The phenotype data supporting the conclusions of this article are only available on request from the BfS server located MEDEORA databank. The genotype data are available on request from the STORE^DB^. ( https://www.storedb.org/store_v3/study.jsp?studyId=1034 ).
