# Supplementary material for: Gene–radon interaction effects on lung cancer risk: validation of susceptibility loci using archived DNA from German Uranium miners
Source: Genes Environ. 2026 Oct 26;48:17. doi: 10.1186/s41021-026-00365-z (PMC13711865; doi:10.1186/s41021-026-00365-z)
Supplement: Supplementary file 1 [file 41021_2026_365_MOESM1_ESM.pdf]

Gene–Radon Interaction Effects on Lung Cancer Risk: Validation of Susceptibility Loci Using Archived DNA from German Uranium Miners

Rosenberger Albert, Bonzheim Irina, Fend Falko, Hochstrat Beate, Johnen Georg, Brik Alexander, Rozynek Peter, Bickeböller Heike, Mörtl Simone, Gomolka Maria

Contents

Supplementary Information 2
Literature search 2
Genes associated with radon-induced lung cancer in a GWAS 2
Priority list of markers to be typed 3
Examples figures of agarose gel of DNA samples 6
Additional Figures on GxE-interaction with all exposures for selected markers 8
G model: genotypic relative risk (HWE distortion test) 11
G model: genetic relative risk 14
Details to: Translation of the genotype doses into genotype probabilities 14
MCMC estimation 16
References 17

Supplementary Information

Literature search

We searched for specialist publications on the topic of <u>genomic</u> associations in radon-induced lung cancer, with a particular focus on susceptibility genes. We did NOT searches for genes generally associated to lung cancer, DNA repair, cellular or intercellular changes, other radio-biological effects, or radiotherapy efficacy and side effects.
Complex search terms were formulated using the keywords "lung cancer", "radon" or "radiation" and "genetics" in order to identify informative specialist publications. Keywords for exclusion were "*radiotherapy".* The searches were restricted using the following criteria: Year of publication: from 2000 onwards; species: human, publication type: excluding editorials; Disease: lung cancer (including all subclassifications).
The following literature databases were searched: PubMed[1], *EMBASE*[2] and the *Web of Science*[3].

An initial review yielded 697 identified academic publications (554 unique hits, 137 duplicates, 5 triplicates and 1 quintuple). Further identical hits were identified using the reference management programme Zotero. A further five publications on potential susceptibility genes for radon-induced lung cancer were drawn from the review article by Choi et al.. (1). We completed the search by citation tracking. Finally 145 publications were selected with either "DNA", "radon", "radon-induced" or "marker" or "loci" in title or abstract. All these "hits" were manually screened for relevant information (on genomic markers and genes potentially associated with radon-induced lung cancer).

Genes associated with radon-induced lung cancer in a GWAS

In a genome-wide interaction analysis of genetically determined radiation sensitivity in WISMUT miners by Rosenberger et al. (2), nine chromosomal regions with radon-gene interactions were identified using various genetic-epidemiological methods.

Investigated exposure/population and location of the main hits of literature search are listed in S-Table 1. Selected genes associated with radon-induced lung cancer in a GWAS are listed in S-Table 2.

S-Table 1 Main hits of literature search: investigated exposure/population and location

**Author**	***exposure/*** **population**	***location***
**Ruano-Ravina A et al. (2014) (3)**	indoor radon	Galicia (Spain)
**Bonner MR et al.(2006) (4)**	indoor radon	Missouri and Iowa
**Leng S et al.(2016) (5)**	uranium miners	Colorado Plateau. replicated in GENEVA und PLCO
**Su S et al. (2006) (6)**	uranium miners	China
**Leng S et al.(2013) (7)**	uranium miners	Colorado-Plateaus
**Vähäkangas KH (1992) (8)**	radon exp. miners	New Mexico
**Yngveson A (1999) (9)**	indoor radon	Swedish lung cancer cases
**Taylor (1994) (10)**	uranium miners	Colorado Plateau
**Minina et al. (2019) (11)**	residents of coal-mining regions	Kemerovo (Russian Federation)
**Rosenberger et al. (2018) (2)**	uranium miners	WISMUT miners (Germany)

1 https://pubmed.ncbi.nlm.nih.gov/
2 https://www.embase.com/login#
3 https://clarivate.com/webofsciencegroup/solutions/web-of-science/

S-Table 2 Selected genes associated with radon-induced lung cancer in a GWAS

Gene	Chromosome		P-value
**Genome-wide significance (multi-marker models)**			
UBE2U	1p31.3	multi-marker models	$5{,}4x10^{-7}$
LOC107985187, LOC105372156, RP11-325K19.1	18q21.32	multi-marker models	$2{,}1x10^{-7}$
**Identified through gene set analysis**			
FTO/ALKBH9	16q12.2	gene set analysis	0,0130
CUBN	10p13	gene set analysis	$1{,}8x10^{-6}$
SOX5, MIR920	12p12.1	gene set analysis	$9{,}5x10^{-6}$
**Suggestive significance (single- or multi-marker models)**			
CSNK1G3, LINCO1170	5q23.2	single-marker models	$2{,}5x10^{-6}$
NAV2, NAV2AS4, NAV2AS5	11p15.1	multi-marker models	4×10^{-6}
CD163L1, ACSM4, PEX5	12p13.31	multi-marker models	4×10^{-6}
C15orf32, ST8SIA2, snoU109	15q26.1	multi-marker models	4×10^{-6}

Priority list of markers to be typed

Based on an extensive literature search, a list of 34 genes was compiled, evaluated and prioritised according to association, function and expression (12–15). These genes were either associated with radon-induced LC, known as lung-cancer risk-genes or considered relevant to the lung-cancer mechanism.

Prioritisation was based on the following score (simplified):

$$score \; = \; b_{typ\ of\ gene} b_{exposition} \; * \; (w_1 b_1 * func. + w_2 b_2 * expr. + w_3 b_3 * asso.)$$

where $b_{type\ of\ gene}$ is a multiplier to distinguish lncRNA, miRNA, novel snoRNA, processed pseudogenes and protein-coding genes, $b_{exposure}$ is a multiplier for exposure group (occupational, indoor-radon exposure) and w_1-w_3 are weights of the assessment categories ($\sum w_1 = 1$), b_1-b_3 are scores of individual criteria of the assessment categories. All parameter values used are factually justified but to a certain extend subjective.

The gene function was categorised as either unknown, lung- or cancer-relevant, irrelevant or multiple/global. The gene expression was considered as unknown, moderately in the lung, primarily in other organs/tissues, multiplayer, primarily in the lung. The gene association was categorised as none, yes – detected by a GWAS, yes – detected not in a GWAS.

Weights and multipliers used for gene prioritisation are given in S-Table 3.

Genes ranking after prioritisation is given in S-Table 4 along with literature references.

S-Table 3 (subjective) weights and multiplier used for gene prioritisation

**function**	*weights w*	**expression**	*weights w*
unknown / non-specific	*0*	unknown	*0*
lung/cancer-relevant	*1*	moderate in Lung	*0,33*
irrelevant	*0,5*	other organs/tissues	*0,67*
multiple/global	*0,75*	multiplayer[$]	*0,75*
		primarily the lungs	*1*

**association with**	**other cancer entity**	**lung cancer**	**others**	**phenotypes**
	weights w	*weights w*		*weights w*
no	*0*	*0*	no	*0*
yes (not GWAS)	*0,375*	*0,75*	indirect (not GWAS)	*0,5*
yes	*0,5*	*1*	indirect-smoking	*0,75*
multiple	*1*	--	yes	*1*

**p-value Interaction**	**in referenced study**	*weights w*	**in Rosenberger et al. (2018)**	*weights w*
	gw[§]-siginificant	*1*	*** significant (≤0,0001)	*1*
	gw[§]-suggestive	*0,75*	** significant (≤0,001)	*0,75*
	significant	*0,5*	* significant (≤0,01)	*0,5*
	not significant	*0,25*	significant (≤0,05)	*0,25*
	>0,5	*0*	not significant	*0*

[§] genome wide, [$] ubiquitary expression

**studied population / exposure**	*multiplier b*	**type of gene**	*multiplier b*
both	*3*	lncRNA	*0,1*
miner	*3*	miRNA	*0,5*
indoor radon	*2,5*	novel snoRNA	*0,1*
surrounding area	*1*	processed pseudogene	*0*
		protein coding	*1*

S-Table 4 List of 34 genes reported as associated with radon-induced lung cancer

rank	ref.	gene	cytogenetic location	studied population / exposure	association with			p-value Interaction$	function	expression	score
					lung cancer	other cancer entity	others phenotypes				
1	(2)	FTO	16q12.2	miners	no	multiple	yes	0.03045	multiple	multiplayer	0.48
2	(2,16)	UBE2U	1p31.3	miners	no	yes	no	0.000003	multiple	other organs/tissues	0.47
3	(2)	CUBN	10p13	miners	no	yes	no	0.00016	lung/cancer-relevant	other organs/tissues	0.44
4	(2)	ST8SIA2	15q26.1	miners	no	yes	indirect-smoking	0.00738	non-specific	other organs/tissues	0.35
4	(2)	SOX5	12p12.1	miners	no	yes	yes	0.41616	multiple	other organs/tissues	0.35
6	(1,8–10)	TP53	17p13.1	both	no	multiple	no	0.18212	lung/cancer-relevant	other organs/tissues	0.33
7	(2)	CD163L1	12p13.31	miners	no	no	no	0.00099	irrelevant	other organs/tissues	0.32
8	(1,6)	CDKN2A	9p21.3	Nuclear workers	yes	multiple	no	0.25715	lung/cancer-relevant	other organs/tissues	0.29
9	(2)	NAV2	11p15.1	miners	no	no	no	0.00390	irrelevant	multiplayer	0.27
10	(1,7)	SIRT1	10q21.3	miners	no	no	indirect (not GWAS)	0.94078	multiple/global	multiplayer	0.24
11	(3)	EPHX1	1q42.12	Indoor radon	no	no	indirect-smoking	0.98877	lung/cancer-relevant	other organs/tissues	0.23
12	(1,3)	GSTT1	22q11.23	Indoor radon	no	yes (not GWAS)	indirect-smoking	0.93947	irrelevant	other organs/tissues	0.22
13	(3)	GSTT2	22q11.23	Indoor radon	no	no	indirect-smoking	0.28237	irrelevant	other organs/tissues	0.18
14	(2)	CSNK1G3	5q23.2	miners	no	no	no	0.06614	multiple/global	multiplayer	0.18
15	(6)	MGMT	10q26.3	Indoor radon	no	no	indirect-smoking	0.51581	multiple/global	moderate in lung	0.17
16	(1,11,17)	ERCC5 (XPG)	13q33.1	Indoor radon	no	no	no	0.85148	lung/cancer-relevant	multiplayer	0.16
16	(1,4)	GSTM1	1p13.3	Indoor radon	no	yes	no	0.31323	irrelevant	multiplayer	0.16
18	(1,5)	IL6	7p15.3	miners	no	no	no	0.20946	lung/cancer-relevant	moderate in lung	0.16
19	(2)	PEX5	12p13.31	miners	no	no	no	0.35559	irrelevant	multiplayer	0.15
20	(2)	MIR920	12p12.1	miners	no	no	no	0.41616	multiple/global	other organs/tissues	0.08
21	(11,18,19)	ATM	11q22.3	surrounding area	yes	multiple	no	0.86709	lung/cancer-relevant	multiplayer	0.06
22	(2)	ACSM4	12p13.31	miners	no	no	no	0.35559	irrelevant	unknown	0.06
23	(2)	LINC01170	5q23.2	miners	no	yes	yes	0.00016	unknown/non-specific	other organs/tissues	0.04
24	(1,11,20–23)	XRCC1	19q13.31	surrounding area	no	yes	no	0.97575	lung/cancer-relevant	multiplayer	0.03
25	(1,17,23)	PARP1	1q42.12	surrounding area	no	yes	no	0.99969	multiple/global	multiplayer	0.03
26	(2)	C15orf32	15q26.1	miners	no	no	yes	0.00738	unknown/non-specific	unknown	0.02
27	(1,11,23–26)	OGG1	3p25.3	surrounding area	no	yes (not GWAS)	no	0.16231	multiple/global	multiplayer	0.02
28	(1,11,23)	ERCC2 (XPD)	19q13.32	surrounding area	no	no	no	0.10883	lung/cancer-relevant	multiplayer	0.02
28	(1,11,17,23)	NBN (NBS1)	8q21.3	surrounding area	no	no	no	0.26233	lung/cancer-relevant	multiplayer	0.02
28	(11,23,27)	APEX1	14q11.2	surrounding area	no	no	no	0.31273	lung/cancer-relevant	multiplayer	0.02
31	(2)	snoU109.3-201	15q26.1	miners	no	no	no	0.00738	multiple/global	unknown	0.02
32	(2)	NAV2-AS4	11p15.1	miners	no	no	no	0.00390	unknown/non-specific	unknown	0.01
32	(2)	NAV2-AS5	11p15.1	miners	no	no	no	0.00390	unknown/non-specific	unknown	0.01
34	(2)	CTBP2P3	18q21.32	miners	no	no	no	1.00000	unknown/non-specific	unknown	0.00

$ in Rosenberger et al. (2018). Genetic modifiers of radon-induced lung cancer risk: a genome-wide interaction study in former uranium miners. (2)

Examples figures of agarose gel of DNA samples

Representative agarose gel of DNA samples isolated from FFPE tissue. M denotes the molecular weight standard Lambda DNA/HindIII.

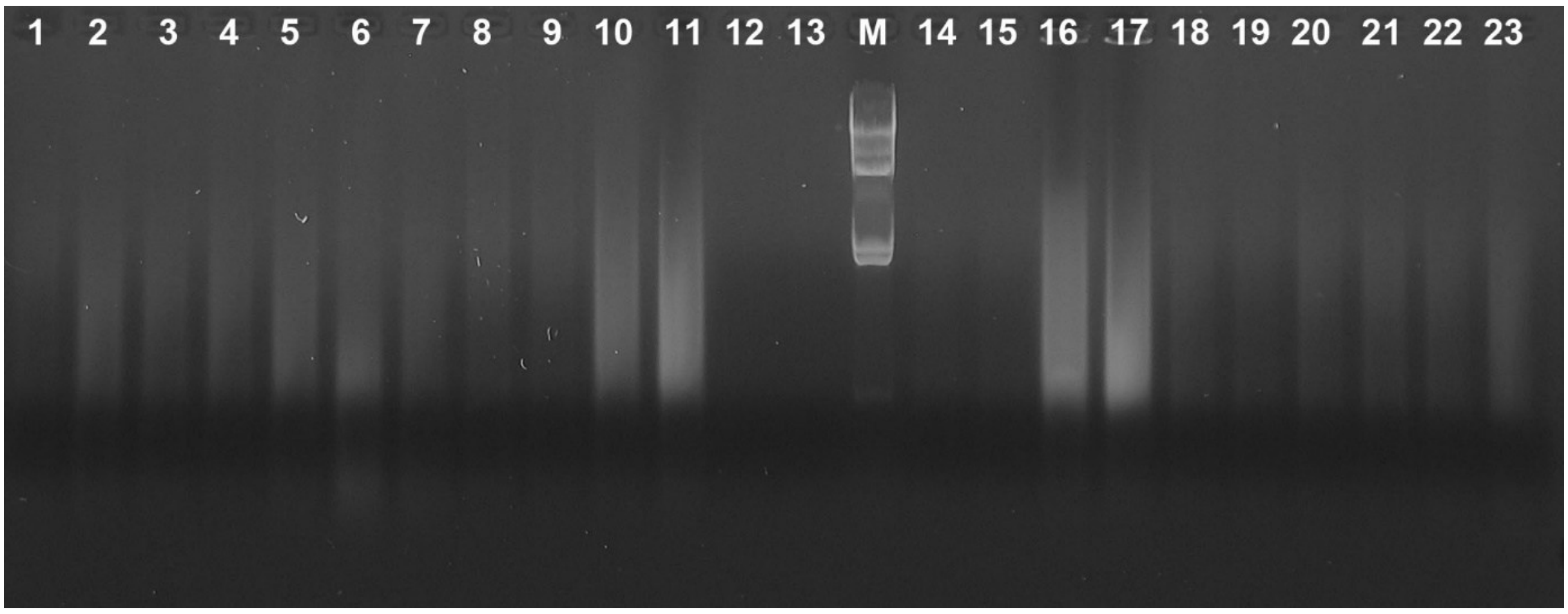

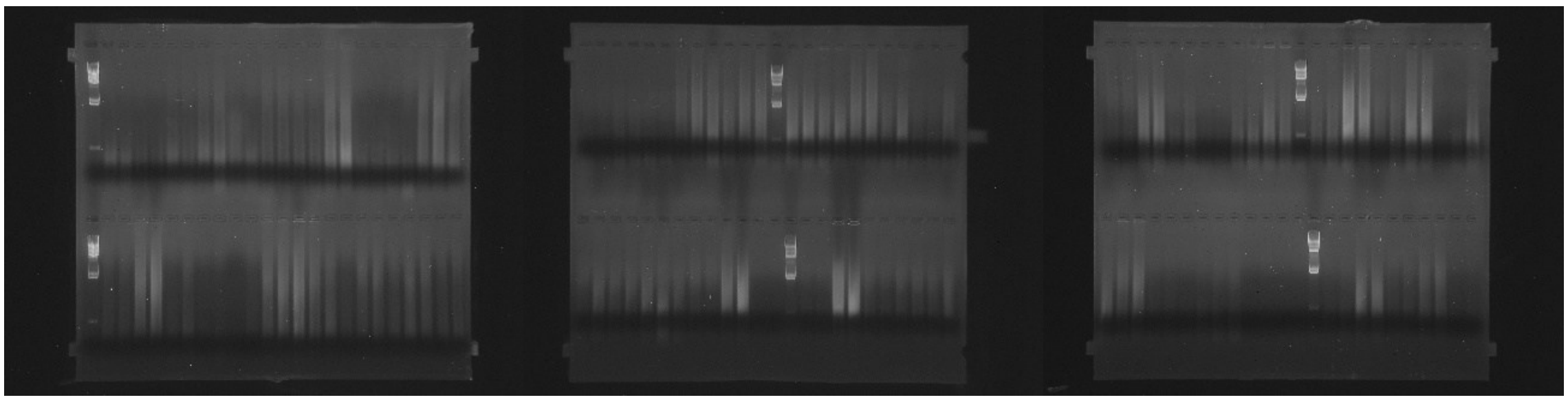

Amplicons designed for the ApliSeq Custom Panel

The following short length amplicons were used for the custom panel of 29 selected markers, optimized for highly degraded DNA and designed with the Ampliseq Designer of Thermo Fisher Scientific (Waltham, MA, USA):

S-Table 5 Amplicons from the AmpliSeq Custom Panel showing the gene regions covered (the primer sequences located 5' and 3' of the amplicons are not specified by the manufacturer).

Gen	Chromosome	Genom. Pos. 5'	Genom. Pos. 3'	Amplikon-ID
UBE2U	chr1	64664984	64665056	AMPL7170886941
UBE2U	chr1	64683980	64684056	AMPL7173964127
UBE2U	chr1	64804825	64804909	AMPL7173964110
UBE2U	chr1	64805442	64805533	AMPL7173964132
CDKN2A	chr9	21816525	21816603	AMPL7173964126
CDKN2A	chr9	21984623	21984708	AMPL7173964114
CDKN2A	chr9	22081743	22081822	AMPL7173964121
CUBN	chr10	17005937	17006023	AMPL7173964120
CUBN	chr10	17046559	17046648	AMPL7173964125
CUBN	chr10	17250834	17250919	AMPL7173964115
SIRT1	chr10	69670762	69670845	AMPL7173964134
SIRT1	chr10	69713245	69713324	AMPL7173964129
NAV2	chr11	19265166	19265253	AMPL7173964124
NAV2	chr11	19466809	19466897	AMPL7173964111
NAV2	chr11	19468732	19468818	AMPL7169717107
CD163L1	chr12	7382167	7382255	AMPL7173964116
CD163L1	chr12	7451765	7451852	AMPL7173964109
CD163L1	chr12	7499433	7499524	AMPL7173964128
SOX5	chr12	24190966	24191052	AMPL7173964131
SOX5	chr12	24244718	24244785	AMPL7173964108
ST8SIA2	chr15	92898556	92898627	AMPL7173964123
ST8SIA2	chr15	92988723	92988811	AMPL7173964118
ST8SIA2	chr15	93006043	93006133	AMPL7173964113
FTO	chr16	53860978	53861057	AMPL7173964133
FTO	chr16	53988937	53989026	AMPL7173964117
FTO	chr16	54000861	54000945	AMPL7173964130
TP53	chr17	7592771	7592854	AMPL7173964112
TP53	chr17	7614592	7614674	AMPL7173964119
TP53	chr17	7711092	7711184	AMPL7173964122

Genom. Pos. according to Human Build GRCh37

Additional Figures on GxE-interaction with all exposures for selected markers

Significant GxE interaction with all occupational exposures is presented in S-Table 6 (p-values, as given in Tables 5 of the main text) and in S-Figure 1. Please note: Horizontal lines indicate that there is no GxE interaction, as the genetic relative risk (RR) is the same for all exposure levels. For the sake of comparability, the quantiles of the exposures are plotted on the x-axis instead of individual values.

S-Table 6: Excerpt from Table 5: Significance of GxE interaction by exposure

Marker	Gene	radon and its decay products [WLM]	LLR [kBq·h/m³]	gamma radiation [mSv]	arsenic [µg/m³·years]	particulate matter [mg/m³·Jahre]	silica-dust [mg/m³·years]
rs7545208	UBE2U	**0.0187**	0.2697	0.4950	0.7034	0.6328	0.5785
rs10965227	CDKN2A	**0.0252**	0.4182	0.3781	0.0897	0.5800	0.3674
rs10762183	SIRT1	**0.0014**	0.1031	0.2757	0.1455	0.2896	0.0955
rs4756999	NAV2	**0.0002**	0.1085	0.0712	0.8659	0.3298	0.4740
rs1353793	SOX5	0.0813	0.6878	0.4794	**0.0231**	0.8948	0.7339
rs4777965	ST8SIA2	**0.0046**	0.2791	0.3009	**0.0144**	0.4123	0.2811
rs1455777	ST8SIA2	**0.0003**	0.2940	0.3742	0.0551	0.4457	0.0618
rs3784722	ST8SIA2	0.3993	**0.0191**	**0.0005**	0.0850	0.2331	0.1816
rs7221056	TP53	**0.0303**	0.1427	0.2613	0.2761	0.3669	0.3049

ŧ for rs7545208 a p-value of 0.0187 was observed considering WLM, when fixing τ at 1; LLR long-lived radionuclides

S-Figure 1: GxE-interaction with all exposures for selected markers

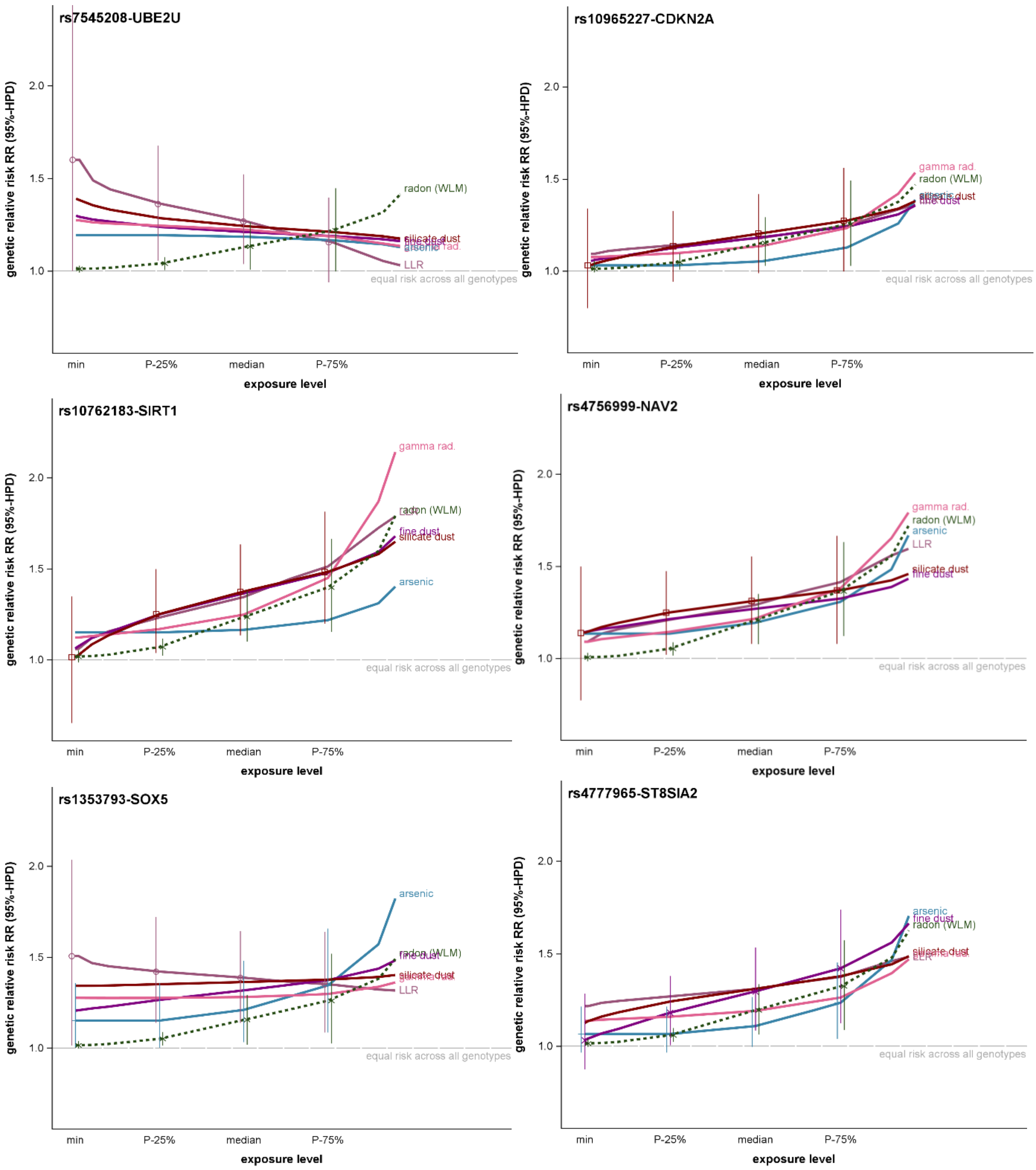

S-Figure 1 (cont.): GxE-interaction with all exposures for selected markers

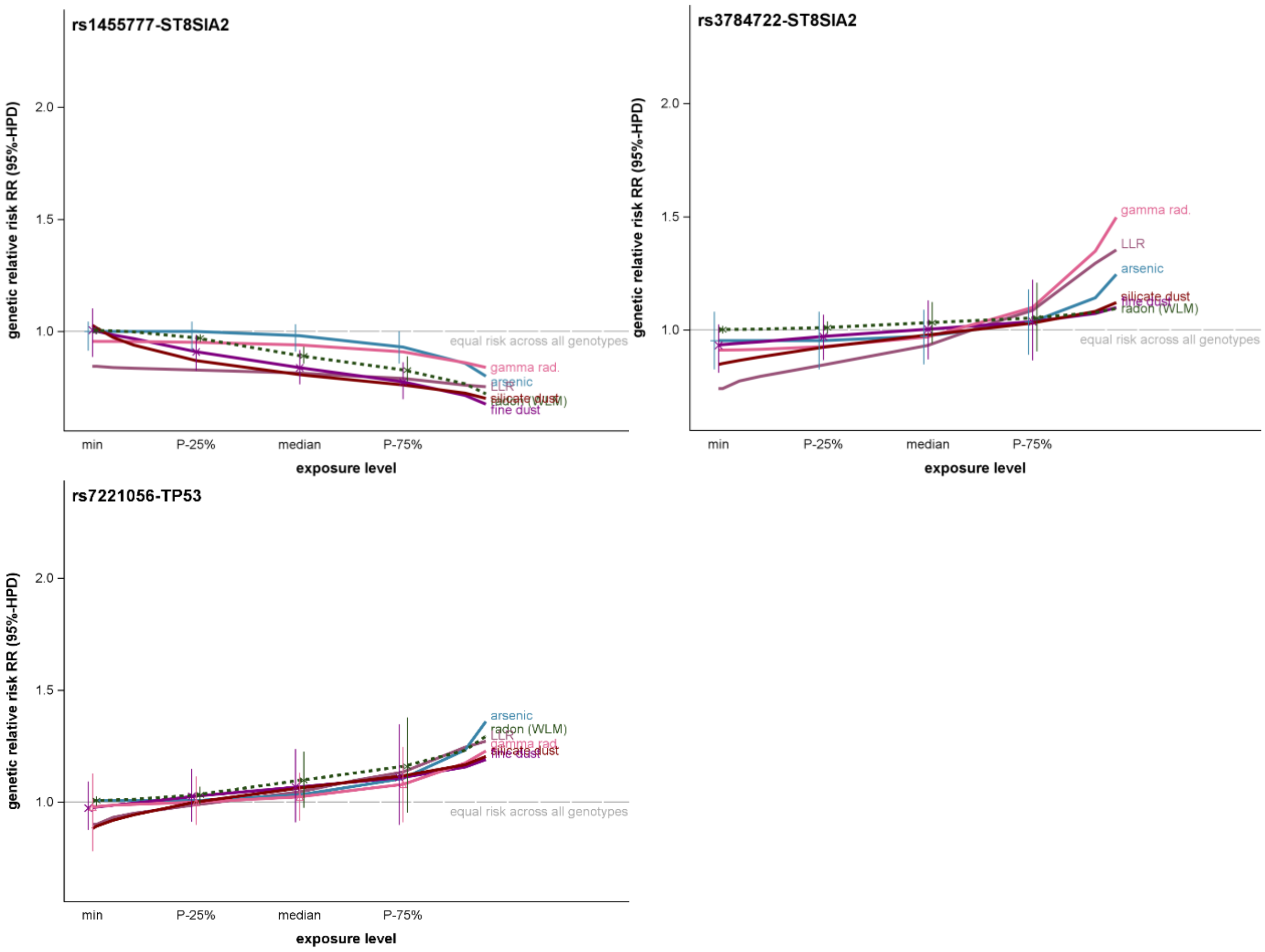

A relative genetic risk of RR=1 means that all genotypes have the same genetic RR. Horizontal curve gradients would indicate that the relative genetic risk RR does NOT change with the degree of exposure and therefore there is no GxE interaction. In a pure GxE interaction, the genetic relative risk should be 1 at zero exposure (starting point of the line). For visual comparison, the exposures were scaled so that the quartiles are positioned identically. The minimum and the 25%, 50% and 75% percentiles are highlighted on the x-axis. The curves for each exposure were scaled so that the percentiles correspond graphically.

G model: genotypic relative risk (HWE distortion test)

The G model essentially describes the distribution of genotypes $[g_0 \quad g_1 \quad g_2]$ among "cases", taking into account the minor allele frequency f (MAF), the genetic relative risk (RR), with F also allowing a deviation from g_1 (excess heterozygosity). The distribution of genotypes in the source population is assumed to follow the HWE. It is given in S-Table 7 Genotype distribution among affected individuals (cases, patients) based on the HWE

We have taken uncertain genotypes into account by weighting them according to genotype probabilities (see Details to: Translation of the genotype doses into genotype probabilities).

S-Table 7 Genotype distribution among affected individuals (cases, patients) based on the HWE

genotypes g (minor allele count)	0	1	2
Observed proportion of the genotype	$g_0 = {}^{n_0}/_n$	$g_{1={}^{n_1}/_n}$	$g_{2={}^{n_1}/_n}$
$P_{g,HWE}$: fraction of genotypes assuming HWE	$(1-f)^2$	$2f(1-f)$	f^2
relative penetrance*	1	RR	$2*(RR-1)+1 = 2RR-1$
risk of illness	p	$p \cdot RR$	$p \cdot (2RR-1)$
$P_{g,HWE,affected}$: fraction of those affected in the total population	$p \cdot (1-f)^2$	$p \cdot RR \cdot 2f(1-f)$	$p \cdot (2RR-1).f^2$
$P_{HWE,affected}$ Proportion of people with the disease in the total population		$p\left\{\begin{matrix}(1-f)^2 + \\ RR \cdot 2f(1-f) + \\ (2RR-1) \cdot f^2\end{matrix}\right\}$	
Proportion of g among those affected: $P_{g,HWE} \mid affected$ (in short e_g	$e_0 = \frac{\not{p} \cdot (1-f)^2}{\not{p}\{\dots\}}$	$e_1 = \frac{\not{p} \cdot RR \cdot 2f(1-f)}{\not{p}\{\dots\}}$	$e_2 = \frac{\not{p}(2RR-1).f^2}{\not{p}\{\dots\}}$

n_0, n_1, n_2 are the numbers of individuals carrying 0, 1 or 2 copies of the rare allele. $n = n_0 + n_1 + n_2$ is the sample size. In case of uncertain genotyping and $r_{0,i}, r_{1,i}, r_{2,i}$ indicating the genotype probabilities of individual i: $n_0 = \sum_{i=1}^{n} r_{0,i}$. The same applies for n_1 and n_2.
* *Penetrance* p: fraction of individuals with a specific genotype g who express the expected phenotype *(here: lung cancer). Across all genotypes,* this is equivalent to *incidence* or *risk of disease*; f minor allele frequency (MAF), RR genetic relative risk

However, there are also mechanisms that can lead to a heterozygous or homozygous excess relative to HWE. Reasons for a heterozygote excess may include: e.g., a) hetero-positive selection (heterozygous individuals have a higher probability of survival or reproduction); b) Wahlund effect (mixing of genetically inhomogeneous subpopulations); c) negative assortative mating (individuals prefer to choose partners who are genetically different from themselves). Reasons for a homozygous excess may include: e.g., e) hidden relationships (hidden relationships or concealed population structures); f) inbreeding (consanguinity: the mating of individuals who are related in both the narrow and a broader sense); g) positive assortative mating (individuals preferentially choose partners with similar characteristics and consequently similar genotypes); h) homo-positive selection (when homozygous individuals have a higher probability of survival or reproduction).

Against the backdrop of possible inbreeding, a distribution of the genotype of a diallelic genomic marker with allele frequency f (extended HWE) had to be defined as given in S-Table 8 (28). The proportion of g among those affected ($e_g = P_{g,HWE} \mid affected$) changes accordingly.

S-Table 8 Extended HWE

genotypes g	0	1	2
$P_{g,extHWE}$: fraction of genotypes assuming extended HWE	$(1-f)^2(1-F)+F(1-f)$	$2f(1-f)(1-F)$	$f^2(1-F)+fF$
$P_{HWE,affected}$ Proportion of people with the disease in the total population	$\left\{\begin{matrix}(1-f)^2(1-F)+F(1-f)+\\ RR\cdot(2f(1-f)(1-F))+\\ (2RR-1)\cdot(f^2(1-F)+fF)\end{matrix}\right\}$		

F excess homozygosity

The likelihood function L is used as the basis for the estimation. L is the probability of the observed sample occurring, calculated from the theoretical distribution of the three genotypes described above, given the parameters f, RR and F:

$$L(f,F,RR)=\sum_{g=0}^{2} n_g\cdot e_g(f,F,RR)$$

with n_g the numbers of individuals carrying $g=0,1\ or\ 2$ copies of the rare allele.

The parameter RR is of central interest. It describes the disease risk of carriers of genotype g_1 (heterozygote) or g_2 (homozygote rarer allele) relative to that of carriers of genotype g_0 (homozygote more frequent allele). The last row of S-Table 7 shows that the “proportion of genotype g among those affected” is independent of *risk of disease* p.

The parameter *RR* can be estimated by maximising the likelihood of observed genotypes $[g_0\quad g_1\quad g_2]$, given MAF and F as known. Since the likelihood function might not be unimodal (as shown in S-Table 9), the value of *f* (MAF) was set to the value given for persons of European descent in publicly accessible databases.

S-Table 9 Likelihood-function of two fictive autosomal markers

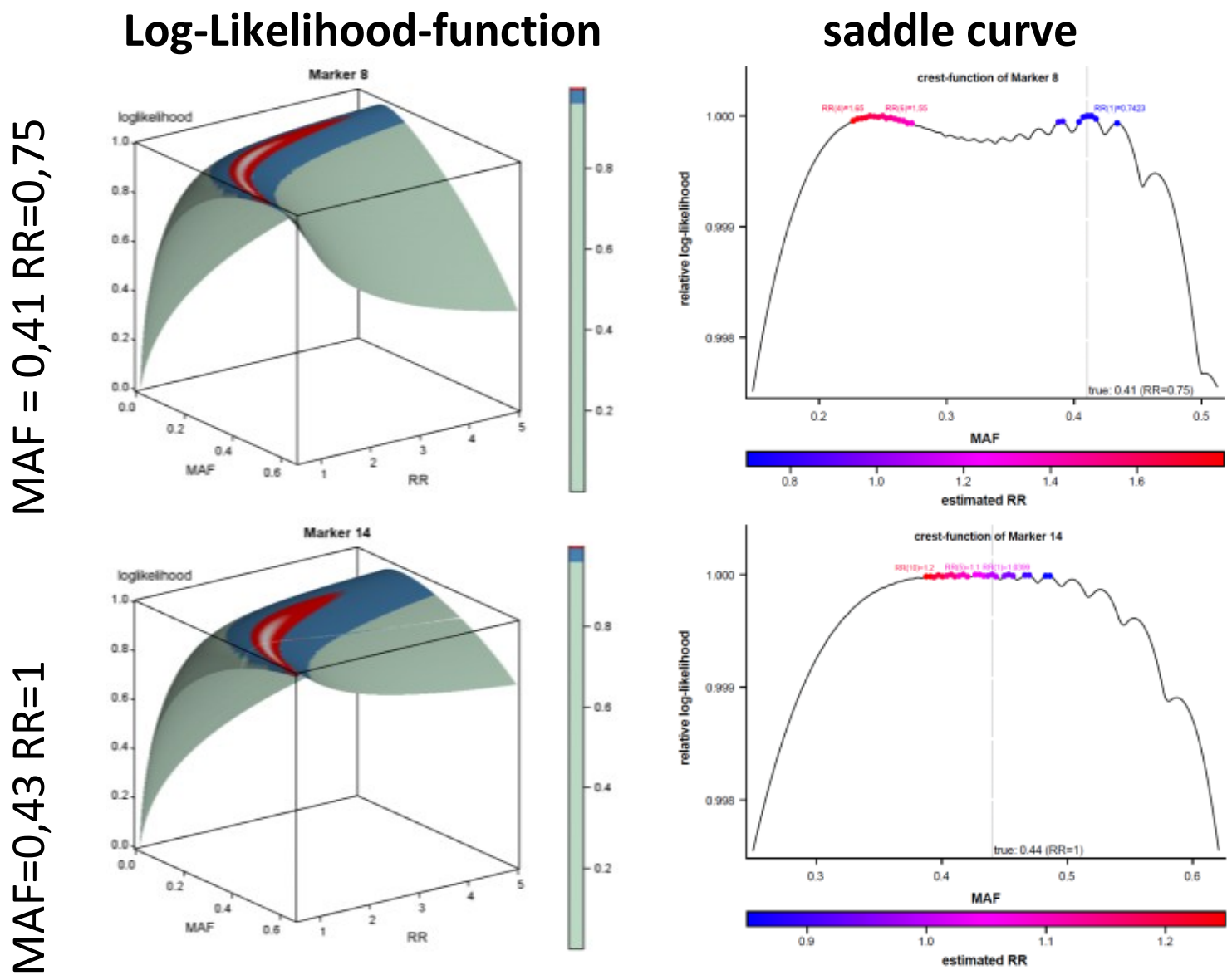

In case for MAF and F only an a-priori distribution can be assumed, the joint posterior distribution of parameters *f, RR* and *F* of the G model (likelihood x prior distributions of the parameters) can be mapped within the Bayesian MCMC framework. Since the likelihood function might not be unimodal, an informative a-posteriori distribution of *f* (MAF), around the value given for persons of European descent in publicly accessible databases, is required.

We used the following a-priori distribution:

f (MAF): *informative*: For a sample of *k* individuals (2*500=1,000 alleles), the number of risk alleles follows a binomial distribution: $x \sim binom(\pi = MAFpop, n = 2k)$. The sample MAF is proportional by $MAF = x/2k$. The expected value of MAF is μ= MAF_{pop}, (population MAF for persons of European descent); the variance $\sigma_{\mu}^2 = \mu \frac{1-\mu}{n}$.
Hence, a beta distribution $B(\alpha, \beta)$, i.e. the conjugate prior probability distribution of the binomial distribution, was used as the prior distribution of the MAF. Their parameters α and β were chosen so that μ and σ_{μ}^2 remain unchanged.

RR: *non-informative: log-normal distribution* $logN(\mathbf{0}, \mathbf{20})$,
restricting RR to positive values with an expected value of E(RR)=1

F: *restrictive, non-information:* uniform distribution $U(-0.1; +0,1)$

G model: genetic relative risk

S-Table 10 G Model: MCMC estimates from MAF and RR

Marker	Gen	MAF	95%-HDI	RR	95%-HDI	p_{direkt}[†]
rs7545208	*UBE2U*	0.34	0.31-0.36	1.25	1.02-1.46	0.0135
rs7526950	*UBE2U*	0.23	0.21-0.25	1.20	1.02-1.35	0.0325
rs10889478	*UBE2U*	0.48	0.46-0.51	0.95	0.82-1.08	0.5181
rs1908635	*UBE2U*	0.17	0.16-0.19	1.15	0.96-1.31	0.1152
rs7023680	*CDKN2A*	0.47	0.45-0.49	1.12	0.93-1.29	0.2289
rs3731217	*CDKN2A*	0.14	0.12-0.16	1.15	0.87-1.59	0.4889
rs10965227	*CDKN2A*	0.24	0.22-0.25	1.14	1.03-1.21	0.0276
rs73596224	*CUBN*	0.06	0.06-0.07	1.35	1.10-1.54	0.0073
rs60918373	*CUBN*	0.15	0.13-0.17	1.10	0.89-1.31	0.3133
rs7095324	*CUBN*	0.36	0.34-0.39	1.01	0.86-1.16	0.9439
rs10823112	*SIRT1*	0.06	0.05-0.07	1.22	0.99-1.47	0.0597
rs10762183	*SIRT1*	0.32	0.30-0.34	1.38	1.17-1.61	<0.0001
rs7129281	*NAV2*	0.27	0.25-0.29	0.95	0.74-1.13	0.549
rs11025138	*NAV2*	0.30	0.28-0.32	0.92	0.75-1.07	0.3479
rs4756999	*NAV2*	0.34	0.31-0.36	1.30	1.05-1.52	0.0056
rs7137426	*CD163L1*	0.37	0.35-0.39	1.01	0.86-1.15	0.9482
rs7970379	*CD163L1*	0.05	0.04-0.06	0.89	0.58-1.26	0.502
rs7302538	*CD163L1*/ACSM4	0.06	0.04-0.07	1.18	0.86-1.65	0.517
rs1353793	*SOX5*	0.45	0.43-0.47	1.34	1.14-1.54	<0.0001
rs7978583	*SOX5*	0.46	0.44-0.49	1.03	0.88-1.21	0.7345
rs4777965	*ST8SIA2*	0.38	0.36-0.41	1.30	1.08-1.55	0.0083
rs1455777	*ST8SIA2*	0.50	0.47-0.52	0.80	0.70-0.90	0.0063
rs3784722	*ST8SIA2*	0.37	0.34-0.39	0.97	0.83-1.14	0.7011
rs8061518	*FTO*	0.34	0.32-0.36	1.27	1.07-1.50	0.01
rs6499657	*FTO*	0.16	0.14-0.17	1.22	1.00-1.55	0.091
rs1111483	*FTO*	0.48	0.46-0.51	1.02	0.89-1.18	0.9023
rs17886268	*TP53*	0.04	0.03-0.05	1.14	0.93-1.35	0.3467
rs7141	*TP53*	0.37	0.34-0.39	1.18	0.94-1.43	0.0726
rs7221056	*TP53*	0.33	0.31-0.36	1.05	0.87-1.24	0.6576

[†] H_0: RR=1, MCMC … Markov Chain Monte Carlo

Details to: Translation of the genotype doses into genotype probabilities

FLDSf is a highly flexible six-parametric function for isotonic transformation of a variable X of the value space [0-1] to a variable Y of the same value space [0-1] (29). We applied it to translate observed genotype doses (range 0 to 100) to genotype probabilities (range 0 to 1). The parameters of the FLDSf transformation per marker and per NGS setting were estimated during fitting the G model by maximizing the probability (ML method, with fixed MAF). The 87 FLDSf parameters sets (29 markers x 3 NGS-settings), grouped into 12 clusters with strong similarity (nearest centroid sorting via PROC FASTCLUS, SAS©). FLDSf parameter sets were created.

It is worth to note, that there was no marker and no NGS setting for which one and the same cluster could be assigned. No pattern whatsoever was identified for which NGS setting or which marker a particular FLDSf transformation was considered "optimal".

The genotype probability was then determined from the observed genotype doses (range 0 to 100) according to the related 12 FLDSf transformation clusters, depending on the marker and NGS setting (see S-Figure 2).

S-Figure 2 Twelve clusters of FLDSf transformations

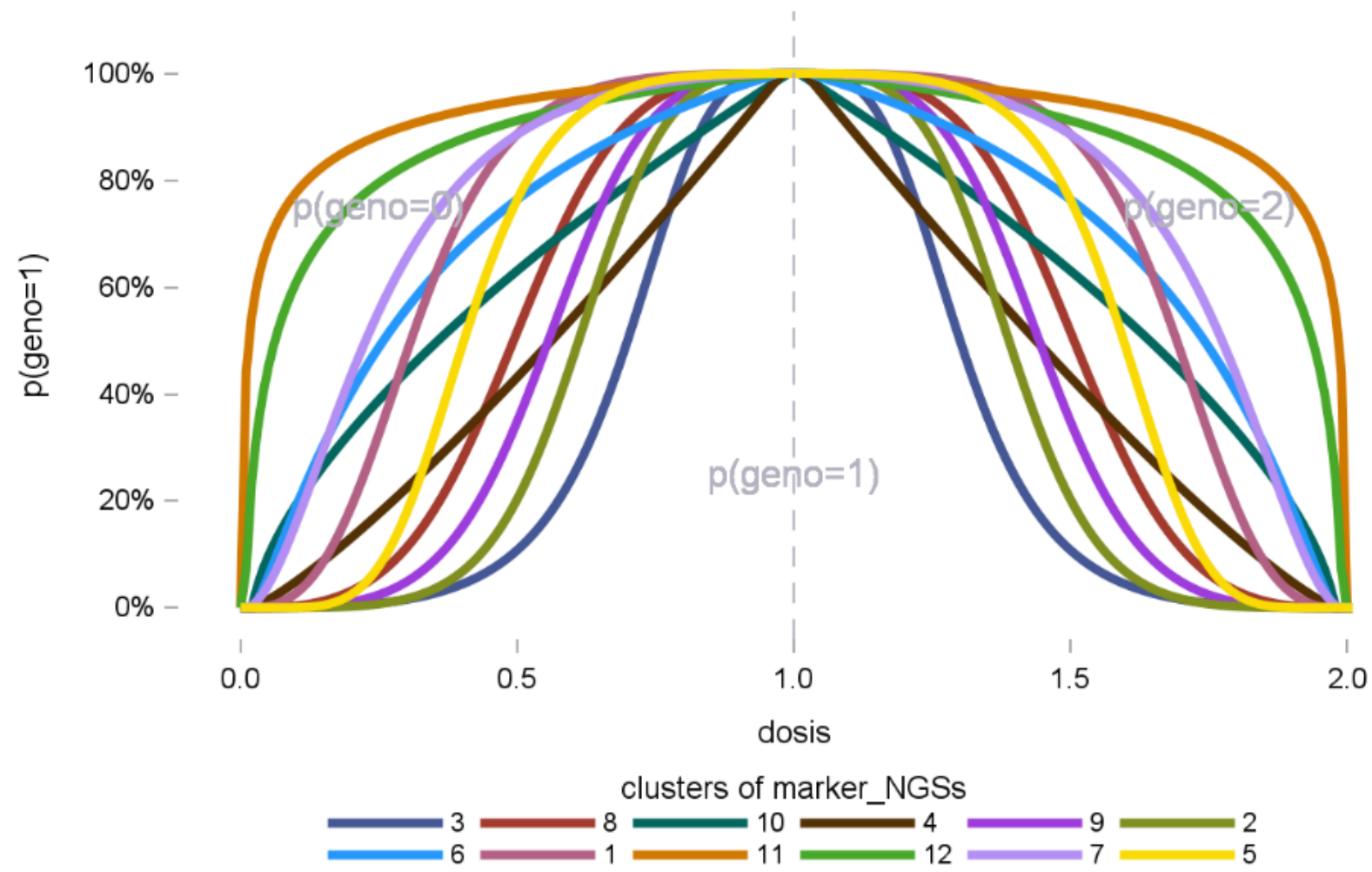

The numbering scheme of “clusters” is none-informative, as the assignment of markers/NGS-settings to clusters.

S-Table 11 Estimated parameters of the twelve clusters of FLDSf transformations

	Function parameter						***Probability of a heterozygosity at dose:***			
Cluster	***a***	***b***	***c***	***d***	***u***	***o***	***10 or 90***	***25 or 75***	***40 or 60***	***F***
3	-2.00	2.50	1.25	1.25	0.02	0.97	1%	15%	96%	0.0100
2	0.40	2.50	1.45	1.00	0.02	0.96	1%	28%	99%	0.0333
9	0.00	2.50	1.25	1.08	0.02	0.97	3%	42%	99%	0.0367
4	0.00	1.00	1.21	1.00	0.02	0.98	20%	48%	87%	0.0271
10	0.00	1.00	0.93	1.43	0.02	0.99	41%	66%	92%	0.0133
8	-0.25	2.50	0.94	1.06	0.03	0.97	11%	67%	100%	0.0375
6	1.46	1.00	1.00	0.77	0.02	0.97	52%	79%	96%	0.0309
5	2.25	2.50	1.13	0.75	0.05	0.97	9%	81%	100%	0.0375
1	1.67	2.50	0.92	1.00	0.02	0.97	38%	91%	100%	0.0333
12	2.00	1.00	0.60	0.70	0.01	0.96	79%	92%	99%	0.0240
7	2.00	1.60	1.00	1.00	0.01	0.97	73%	93%	99%	0.0180
11	2.00	1.00	0.50	1.00	0.00	0.96	89%	96%	99%	0.0240

sorted by dose; F estimated excess heterozygosity compared to HWE

MCMC estimation

The a posteriori distribution of a parameter is calculated using Bayesian methods (MCMC in this work). This reflects the probability of possible parameter values as a function of the observed data and a prior expectation (a priori distribution). Point estimates (here the mean value), interval estimates (here the 95% HDI) and significance measures (here based on the probability of direction) can be derived from the a posteriori distribution (30).

Highest Posterior Density Intervals (HDI)

The 95% HDI is a region with an a posteriori probability of 95%. The minimum density of any point within this region is equal to or greater than the density of any point outside this region.

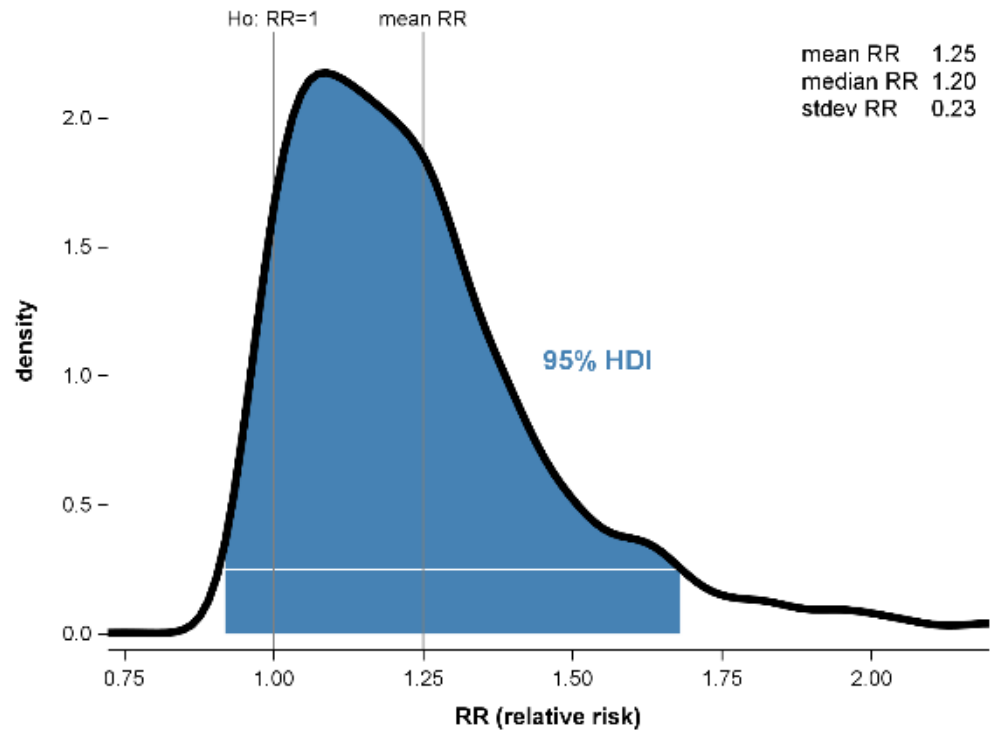

Probability of Direction (p_d)

The probability of direction (p_d) can be defined as the probability that a parameter is smaller (or larger) than a value under the null hypothesis, whichever is the most probable. $(1 - p_d)$ is therefore the equivalent of the one-sided p-value but measured using the a posteriori distribution.

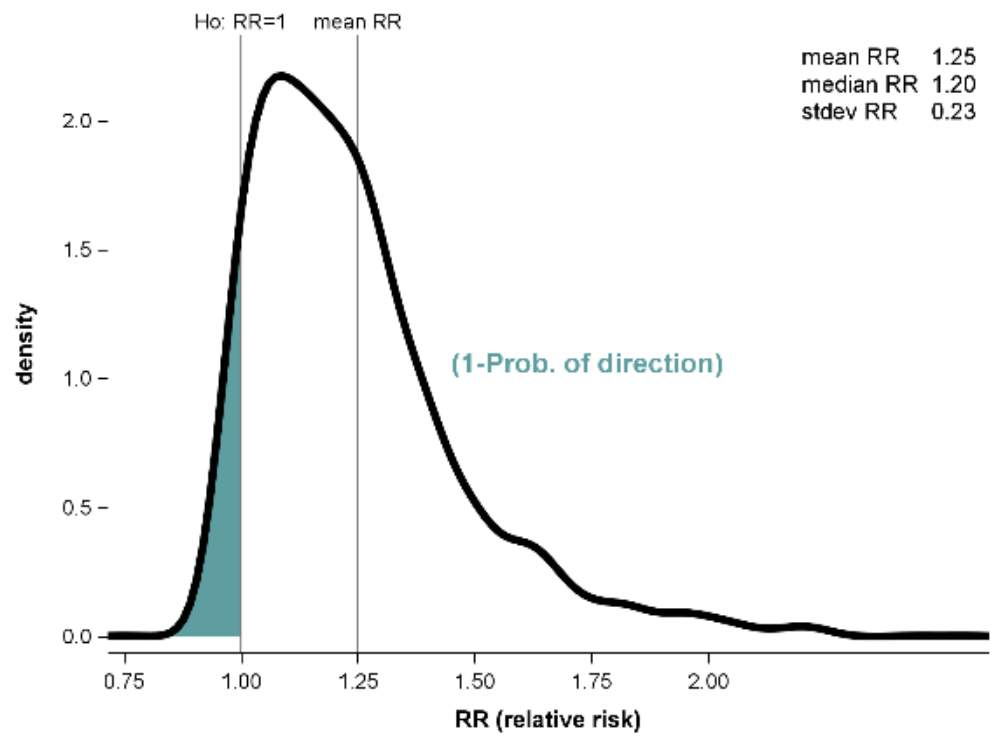

A parameter was labelled as significant in this work if $p_{\text{two-sided}} = 2\times(1-p_d)$ (p_d: “probability of direction”) was lower than 0.05. This corresponds approximately to the p-value of a 2-sided test of frequentist statistics.

References

1. Choi JR, Park SY, Noh OK, Koh YW, Kang DR. Gene mutation discovery research of non-smoking lung cancer patients due to indoor radon exposure. Ann Occup Environ Med. 2016;28:13. Located at: 26985396. doi:10.1186/s40557-016-0095-2

2. Rosenberger A, Hung RJ, Christiani DC, Caporaso NE, Liu G, Bojesen SE, u. a. Genetic modifiers of radon-induced lung cancer risk: a genome-wide interaction study in former uranium miners. Int Arch Occup Environ Health. 3. Juli 2018. Located at: 29971594. doi:10.1007/s00420-018-1334-3

3. Ruano-Ravina A, Pereyra MF, Castro MT, Perez-Rios M, Abal-Arca J, Barros-Dios JM. Genetic susceptibility, residential radon, and lung cancer in a radon prone area. J Thorac Oncol. August 2014;9(8):1073–80. Located at: 24852519. doi:10.1097/JTO.0000000000000205

4. Bonner MR, Bennett WP, Xiong W, Lan Q, Brownson RC, Harris CC, u. a. Radon, secondhand smoke, glutathione-S-transferase M1 and lung cancer among women. Int J Cancer. 15. September 2006;119(6):1462–7. Located at: 16642467. doi:10.1002/ijc.22002

5. Leng S, Thomas CL, Snider AM, Picchi MA, Chen W, Willis DG, u. a. Radon Exposure, IL-6 Promoter Variants, and Lung Squamous Cell Carcinoma in Former Uranium Miners. Environ Health Perspect. April 2016;124(4):445–51. doi:10.1289/ehp.1409437 PubMed PMID: 26372664; PubMed Central PMCID: PMC4829998.

6. Su S, Jin Y, Zhang W, Yang L, Shen Y, Cao Y, u. a. Aberrant Promoter Methylation of p16-INK4a and O-6</sup-Methylguanine-DNA Methyltransferase Genes in Workers at a Chinese Uranium Mine. Journal of Occupational Health. 2006;48(4):261–6. doi:10.1539/joh.48.261

7. Leng S, Picchi MA, Liu Y, Thomas CL, Willis DG, Bernauer AM, u. a. Genetic variation in SIRT1 affects susceptibility of lung squamous cell carcinomas in former uranium miners from the Colorado plateau. Carcinogenesis. Mai 2013;34(5):1044–50. Located at: 23354305. doi:10.1093/carcin/bgt024

8. Vahakangas KH, Samet JM, Metcalf RA, Welsh JA, Bennett WP, Lane DP, u. a. Mutations of p53 and ras genes in radon-associated lung cancer from uranium miners. Lancet [Internet]. März 1992;339(8793):576–80. Located at: 1347094. Verfügbar unter: https://www.ncbi.nlm.nih.gov/pubmed/1347094

9. Yngveson A, Williams C, Hjerpe A, Lundeberg J, Soderkvist P, Pershagen G. p53 Mutations in lung cancer associated with residential radon exposure. Cancer Epidemiol Biomarkers Prev [Internet]. Mai 1999;8(5):433–8. Located at: 10350439. Verfügbar unter: https://www.ncbi.nlm.nih.gov/pubmed/10350439

10. Taylor JA, Watson MA, Devereux TR, Michels RY, Saccomanno G, Anderson M. p53 mutation hotspot in radon-associated lung cancer. Lancet [Internet]. Januar 1994;343(8889):86–7. Located at: 7903781. Verfügbar unter: https://www.ncbi.nlm.nih.gov/pubmed/7903781

11. Minina VI, Bakanova ML, Soboleva OA, Ryzhkova AV, Titov RA, Savchenko YA, u. a. Polymorphisms in DNA repair genes in lung cancer patients living in a coal-mining region. EurJ Cancer Prev. 2019;28(6):522–8. Located at: Embase. doi:10.1097/CEJ.0000000000000504

12. Buniello A, MacArthur JAL, Cerezo M, Harris LW, Hayhurst J, Malangone C, u. a. The NHGRI-EBI GWAS Catalog of published genome-wide association studies, targeted arrays and summary statistics 2019. Nucleic Acids Res. 8. Januar 2019;47(D1):D1005–12. doi:10.1093/nar/gky1120 PubMed PMID: 30445434; PubMed Central PMCID: PMC6323933.

13. National Center for Biotechnology Information (NCBI)[Internet]. Bethesda (MD). dbGene [Internet]. Verfügbar unter: https://www.ncbi.nlm.nih.gov/gene

14. The Human Protein Atlas [Internet]. [zitiert 5. Februar 2021]. Verfügbar unter: https://www.proteinatlas.org/

15. Cunningham F, Amode MR, Barrell D, Beal K, Billis K, Brent S, u. a. Ensembl 2015. Nucleic acids research. Januar 2015;43(Database issue):D662-9. Located at: 25352552. doi:10.1093/nar/gku1010

16. Kazma R, Babron MC, Gaborieau V, Genin E, Brennan P, Hung RJ, u. a. Lung cancer and DNA repair genes: multilevel association analysis from the International Lung Cancer Consortium. Carcinogenesis. 2012/03/03 Aufl. Mai 2012;33(5):1059–64. Located at: 22382497. doi:10.1093/carcin/bgs116

17. Sinitsky MY, Larionov AV, Asanov MA, Druzhinin VG. Associations of DNA-repair gene polymorphisms with a genetic susceptibility to ionizing radiation in residents of areas with high radon (222Rn) concentration. Int J Radiat Biol. Juni 2015;91(6):486–94. doi:10.3109/09553002.2015.1012306 PubMed PMID: 25651041.

18. Byun J, Xiao X, Han Y, Li Y, Sun R, Li X, u. a. Multiethnic genome-wide meta-analysis of 34,329 cases and 35,732 controls identifies cross-ancestry loci for lung cancer susceptibility [Internet]. Proceedings of the Annual Meeting of the American Association for Cancer Research 2020. American Association for Cancer Research; 15. August 2020 [zitiert 26. Oktober 2020]; Philadelphia. Verfügbar unter: https://cancerres.aacrjournals.org/content/80/16_Supplement/3396

19. Schneider J, Illig T, Rosenberger A, Bickeboller H, Wichmann HE. Detection of ATM gene mutations in young lung cancer patients: a population-based control study. Archives of medical research. Februar 2008;39(2):226–31. Located at: 18164969. doi:10.1016/j.arcmed.2007.08.004

20. Sanjari Moghaddam A, Nazarzadeh M, Sanjari Moghaddam H, Bidel Z, Keramatinia A, Darvish H, u. a. XRCC1 Gene Polymorphisms and Breast Cancer Risk: A Systematic Review and Meta- Analysis Study. Asian Pac J Cancer Prev. 2016;17(S3):323–30. doi:10.7314/apjcp.2016.17.s3.323 PubMed PMID: 27165246.

21. Yan J, Wang X, Tao H, Deng Z, Yang W, Lin F. Meta-Analysis of the Relationship between XRCC1-Arg399Gln and Arg280His Polymorphisms and the Risk of Prostate Cancer. Sci Rep. 30. April 2015;5:9905. doi:10.1038/srep09905 PubMed PMID: 25927275; PubMed Central PMCID: PMC4415422.

22. Hornhardt S, Rossler U, Sauter W, Rosenberger A, Illig T, Bickeboller H, u. a. Genetic factors in individual radiation sensitivity. DNA repair. April 2014;16:54–65. Located at: 24674628. doi:10.1016/j.dnarep.2014.02.001

23. Brenner DR, Brennan P, Boffetta P, Amos CI, Spitz MR, Chen C, u. a. Hierarchical modeling identifies novel lung cancer susceptibility variants in inflammation pathways among 10,140 cases and 11,012 controls. Human genetics. 2013/02/02 Aufl. Mai 2013;132(5):579–89. Located at: 23370545. doi:10.1007/s00439-013-1270-y

24. Hung RJ, Christiani DC, Risch A, Popanda O, Haugen A, Zienolddiny S, u. a. International Lung Cancer Consortium: pooled analysis of sequence variants in DNA repair and cell cycle pathways. Cancer epidemiology, biomarkers & prevention : a publication of the American Association for Cancer Research, cosponsored by the American Society of Preventive Oncology. November 2008;17(11):3081–9. Located at: 18990748. doi:10.1158/1055-9965.EPI-08-0411

25. Li H, Hao X, Zhang W, Wei Q, Chen K. The hOGG1 Ser326Cys polymorphism and lung cancer risk: a meta-analysis. Cancer Epidemiol Biomarkers Prev. Juli 2008;17(7):1739–45. doi:10.1158/1055-9965.EPI-08-0001 PubMed PMID: 18628426.

26. Yin ZB, Hua RX, Li JH, Sun C, Zhu JH, Su X, u. a. Smoking and hOGG1 Ser326Cys polymorphism contribute to lung cancer risk: evidence from a meta-analysis. Tumour biology : the journal of the International Society for Oncodevelopmental Biology and Medicine. 2013/10/03 Aufl. Februar 2014;35(2):1609–18. Located at: 24085357. doi:10.1007/s13277-013-1222-0

27. Druzhinin VG, Volkov AN, Glushkov AN, Golovina TA, Minina VI, Ingel' FI, u. a. [Role of repair gene polymorphism in estimating the sensitivity of human genome to radon in concentrations exceeding maximum permissible level]. Gig Sanit. Oktober 2011;(5):26–30. PubMed PMID: 22184996.

28. Cavalli-Sforza LL, Bodmer WF. The Genetics of Human Populations. Mineola, New York: Dover Publications; 1999.

29. Rosenberger A. FLDS: A Flexible, Limited, Double-Sigmoid Transmission Function. Journal of Probability and Statistics. 2026;2026(1):6076741. doi:10.1155/jpas/6076741

30. Makowski D, Ben-Shachar MS, Chen SHA, Lüdecke D. Indices of Effect Existence and Significance in the Bayesian Framework. Frontiers in Psychology [Internet]. 2019 [zitiert 1. November 2022];10. Verfügbar unter: https://www.frontiersin.org/articles/10.3389/fpsyg.2019.02767
